# Germline variation at 8q24 and prostate cancer risk in men of European ancestry

**DOI:** 10.1038/s41467-018-06863-1

**Published:** 2018-11-05

**Authors:** Marco Matejcic, Edward J. Saunders, Tokhir Dadaev, Mark N. Brook, Kan Wang, Xin Sheng, Ali Amin Al Olama, Fredrick R. Schumacher, Sue A. Ingles, Koveela Govindasami, Sara Benlloch, Sonja I. Berndt, Demetrius Albanes, Stella Koutros, Kenneth Muir, Victoria L. Stevens, Susan M. Gapstur, Catherine M. Tangen, Jyotsna Batra, Judith Clements, Henrik Gronberg, Nora Pashayan, Johanna Schleutker, Alicja Wolk, Catharine West, Lorelei Mucci, Peter Kraft, Géraldine Cancel-Tassin, Karina D. Sorensen, Lovise Maehle, Eli M. Grindedal, Sara S. Strom, David E. Neal, Freddie C. Hamdy, Jenny L. Donovan, Ruth C. Travis, Robert J. Hamilton, Barry Rosenstein, Yong-Jie Lu, Graham G. Giles, Adam S. Kibel, Ana Vega, Jeanette T. Bensen, Manolis Kogevinas, Kathryn L. Penney, Jong Y. Park, Janet L. Stanford, Cezary Cybulski, Børge G. Nordestgaard, Hermann Brenner, Christiane Maier, Jeri Kim, Manuel R. Teixeira, Susan L. Neuhausen, Kim De Ruyck, Azad Razack, Lisa F. Newcomb, Davor Lessel, Radka Kaneva, Nawaid Usmani, Frank Claessens, Paul A. Townsend, Manuela Gago-Dominguez, Monique J. Roobol, Florence Menegaux, Kay-Tee Khaw, Lisa A. Cannon-Albright, Hardev Pandha, Stephen N. Thibodeau, Daniel J. Schaid, The PRACTICAL (Prostate Cancer Association Group to Investigate Cancer-Associated Alterations in the Genome) Consortium, Brian E. Henderson, Mariana C. Stern, Alison Thwaites, Michelle Guy, Ian Whitmore, Angela Morgan, Cyril Fisher, Steve Hazel, Naomi Livni, Margaret Cook, Laura Fachal, Stephanie Weinstein, Laura E. Beane Freeman, Robert N. Hoover, Mitchell J. Machiela, Artitaya Lophatananon, Brian D. Carter, Phyllis Goodman, Leire Moya, Srilakshmi Srinivasan, Mary-Anne Kedda, Trina Yeadon, Allison Eckert, Martin Eklund, Carin Cavalli-Bjoerkman, Alison M. Dunning, Csilla Sipeky, Niclas Hakansson, Rebecca Elliott, Hardeep Ranu, Edward Giovannucci, Constance Turman, David J. Hunter, Olivier Cussenot, Torben Falck Orntoft, Athene Lane, Sarah J. Lewis, Michael Davis, Tim J. Key, Paul Brown, Girish S. Kulkarni, Alexandre R. Zlotta, Neil E. Fleshner, Antonio Finelli, Xueying Mao, Jacek Marzec, Robert J. MacInnis, Roger Milne, John L. Hopper, Miguel Aguado, Mariona Bustamante, Gemma Castaño-Vinyals, Esther Gracia-Lavedan, Lluís Cecchini, Meir Stampfer, Jing Ma, Thomas A. Sellers, Milan S. Geybels, Hyun Park, Babu Zachariah, Suzanne Kolb, Dominika Wokolorczyk, Jan Lubinski, Wojciech Kluzniak, Sune F. Nielsen, Maren Weisher, Katarina Cuk, Walther Vogel, Manuel Luedeke, Christopher J. Logothetis, Paula Paulo, Marta Cardoso, Sofia Maia, Maria P. Silva, Linda Steele, Yuan Chun Ding, Gert De Meerleer, Sofie De Langhe, Hubert Thierens, Jasmine Lim, Meng H. Tan, Aik T. Ong, Daniel W. Lin, Darina Kachakova, Atanaska Mitkova, Vanio Mitev, Matthew Parliament, Guido Jenster, Christopher Bangma, F. H. Schroder, Thérèse Truong, Yves Akoli Koudou, Agnieszka Michael, Andrzej Kierzek, Ami Karlsson, Michael Broms, Huihai Wu, Claire Aukim-Hastie, Lori Tillmans, Shaun Riska, Shannon K. McDonnell, David Dearnaley, Amanda Spurdle, Robert Gardiner, Vanessa Hayes, Lisa Butler, Renea Taylor, Melissa Papargiris, Pamela Saunders, Paula Kujala, Kirsi Talala, Kimmo Taari, Søren Bentzen, Belynda Hicks, Aurelie Vogt, Amy Hutchinson, Angela Cox, Anne George, Ants Toi, Andrew Evans, Theodorus H. van der Kwast, Takashi Imai, Shiro Saito, Shan-Chao Zhao, Guoping Ren, Yangling Zhang, Yongwei Yu, Yudong Wu, Ji Wu, Bo Zhou, John Pedersen, Ramón Lobato-Busto, José Manuel Ruiz-Dominguez, Lourdes Mengual, Antonio Alcaraz, Julio Pow-Sang, Kathleen Herkommer, Aleksandrina Vlahova, Tihomir Dikov, Svetlana Christova, Angel Carracedo, Brigitte Tretarre, Xavier Rebillard, Claire Mulot, Jan Adolfsson, Par Stattin, Jan-Erik Johansson, Richard M. Martin, Ian M. Thompson, Suzanne Chambers, Joanne Aitken, Lisa Horvath, Anne-Maree Haynes, Wayne Tilley, Gail Risbridger, Markus Aly, Tobias Nordström, Paul Pharoah, Teuvo L. J. Tammela, Teemu Murtola, Anssi Auvinen, Neil Burnet, Gill Barnett, Gerald Andriole, Aleksandra Klim, Bettina F. Drake, Michael Borre, Sarah Kerns, Harry Ostrer, Hong-Wei Zhang, Guangwen Cao, Ji Lin, Jin Ling, Meiling Li, Ninghan Feng, Jie Li, Weiyang He, Xin Guo, Zan Sun, Guomin Wang, Jianming Guo, Melissa C. Southey, Liesel M. FitzGerald, Gemma Marsden, Antonio Gómez-Caamaño, Ana Carballo, Paula Peleteiro, Patricia Calvo, Robert Szulkin, Javier Llorca, Trinidad Dierssen-Sotos, Ines Gomez-Acebo, Hui-Yi Lin, Elaine A. Ostrander, Rasmus Bisbjerg, Peter Klarskov, Martin Andreas Røder, Peter Iversen, Bernd Holleczek, Christa Stegmaier, Thomas Schnoeller, Philipp Bohnert, Esther M. John, Piet Ost, Soo-Hwang Teo, Marija Gamulin, Tomislav Kulis, Zeljko Kastelan, Chavdar Slavov, Elenko Popov, Thomas Van den Broeck, Steven Joniau, Samantha Larkin, Jose Esteban Castelao, Maria Elena Martinez, Ron H. N. van Schaik, Jianfeng Xu, Sara Lindström, Elio Riboli, Clare Berry, Afshan Siddiq, Federico Canzian, Laurence N. Kolonel, Loic Le Marchand, Matthew Freedman, Sylvie Cenee, Marie Sanchez, Fredrik Wiklund, Stephen J. Chanock, Douglas F. Easton, Rosalind A. Eeles, Zsofia Kote-Jarai, David V. Conti, Christopher A. Haiman

**Affiliations:** 1 Department of Preventive Medicine, Keck School of Medicine, University of Southern California/Norris Comprehensive Cancer Center, Los Angeles, CA 90033 USA; 2 The Institute of Cancer Research, London, SW7 3RP UK; 3 Centre for Cancer Genetic Epidemiology, Department of Public Health and Primary Care, Strangeways Research Laboratory, University of Cambridge, Cambridge, CB1 8RN UK; 4 Department of Clinical Neurosciences, University of Cambridge, Cambridge, CB2 0QQ UK; 5 Department of Population and Quantitative Health Sciences, Case Western Reserve University, Cleveland, OH 44106-7219 USA; 6 Seidman Cancer Center, University Hospitals, Cleveland, OH 44106 USA; 7 Division of Cancer Epidemiology and Genetics, National Cancer Institute, NIH, Bethesda, MD 20892 USA; 8 Institute of Population Health, University of Manchester, Manchester, M13 9PL UK; 9 Warwick Medical School, University of Warwick, Coventry, CV4 7AL UK; 10 Epidemiology Research Program, American Cancer Society, 250 Williams Street, Atlanta, GA 30303 USA; 11 SWOG Statistical Center, Fred Hutchinson Cancer Research Center, Seattle, WA 98109 USA; 12 Australian Prostate Cancer Research Centre-Qld, Institute of Health and Biomedical Innovation and School of Biomedical Science, Queensland University of Technology, Brisbane, QLD 4059 Australia; 13 Translational Research Institute, Brisbane, QLD 4102 Australia; 14 Department of Medical Epidemiology and Biostatistics, Karolinska Institute, SE-171 77 Stockholm, Sweden; 15 Centre for Cancer Genetic Epidemiology, Department of Oncology, Strangeways Research Laboratory, University of Cambridge, Cambridge, CB1 8RN UK; 16 Department of Applied Health Research, University College London, London, WC1E 7HB UK; 17 Department of Medical Biochemistry and Genetics, Institute of Biomedicine, University of Turku, FI-20014 Turku, Finland; 18 Tyks Microbiology and Genetics, Department of Medical Genetics, Turku University Hospital, 20521 Turku, Finland; 19 BioMediTech, University of Tampere, 33520 Tampere, Finland; 20 Division of Nutritional Epidemiology, Institute of Environmental Medicine, Karolinska Institutet, SE-171 77 Stockholm, Sweden; 21 Division of Cancer Sciences, Manchester Academic Health Science Centre, Radiotherapy Related Research, Manchester NIHR Biomedical Research Centre, The Christie Hospital NHS Foundation Trust, University of Manchester, Manchester, M13 9PL UK; 22 Department of Epidemiology, Harvard School of Public Health, Boston, MA 02115 USA; 23 Program in Genetic Epidemiology and Statistical Genetics, Department of Epidemiology, Harvard T.H. Chan School of Public Health, Boston, MA 02115 USA; 24 GRC N°5 ONCOTYPE-URO, UPMC Univ Paris 06, Tenon Hospital, F-75020 Paris, France; 25 CeRePP, Tenon Hospital, F-75020 Paris, France; 26 Department of Molecular Medicine, Aarhus University Hospital, 8200 Aarhus N, Denmark; 27 Department of Clinical Medicine, Aarhus University, 8200 Aarhus N, Denmark; 28 Department of Medical Genetics, Oslo University Hospital, 0424 Oslo, Norway; 29 Department of Epidemiology, The University of Texas MD Anderson Cancer Center, Houston, TX 77030 USA; 30 Department of Oncology, Addenbrooke’s Hospital, University of Cambridge, Cambridge, CB2 0QQ UK; 31 Cancer Research UK Cambridge Research Institute, Li Ka Shing Centre, Cambridge, CB2 0RE UK; 32 Nuffield Department of Surgical Sciences, University of Oxford, Oxford, OX1 2JD UK; 33 School of Social and Community Medicine, University of Bristol, Canynge Hall, 39 Whatley Road, Bristol, BS8 2PS UK; 34 Cancer Epidemiology, Nuffield Department of Population Health, University of Oxford, Oxford, OX3 7LF UK; 35 Department of Surgical Oncology, Princess Margaret Cancer Centre, Toronto, ON M5G 2M9 Canada; 36 Department of Radiation Oncology, Icahn School of Medicine at Mount Sinai, New York, NY 10029 USA; 37 Department of Genetics and Genomic Sciences, Icahn School of Medicine at Mount Sinai, New York, NY 10029-5674 USA; 38 Centre for Molecular Oncology, John Vane Science Centre, Barts Cancer Institute, Queen Mary University of London, London, EC1M 6BQ UK; 39 Cancer Epidemiology & Intelligence Division, Cancer Council Victoria, Melbourne, VIC 3004 Australia; 40 Centre for Epidemiology and Biostatistics, Melbourne School of Population and Global Health, The University of Melbourne, Melbourne, VIC 3010 Australia; 41 Division of Urologic Surgery, Brigham and Womens Hospital, Boston, MA 02115 USA; 42 Fundación Pública Galega de Medicina Xenómica-SERGAS, Grupo de Medicina Xenómica, CIBERER, IDIS, 15706 Santiago de Compostela, Spain; 43 Department of Epidemiology, Gillings School of Global Public Health, University of North Carolina, Columbia, SC 29208 USA; 44 Centre for Research in Environmental Epidemiology (CREAL), Barcelona Institute for Global Health (ISGlobal), 08003 Barcelona, Spain; 45 CIBER Epidemiología y Salud Pública (CIBERESP), 28029 Madrid, Spain; 46 IMIM (Hospital del Mar Research Institute), 08003 Barcelona, Spain; 47 Universitat Pompeu Fabra (UPF), 08002 Barcelona, Spain; 48 Channing Division of Network Medicine, Department of Medicine, Brigham and Women’s Hospital/Harvard Medical School, Boston, MA 02184 USA; 49 Department of Cancer Epidemiology, Moffitt Cancer Center, Tampa, FL 33612 USA; 50 Division of Public Health Sciences, Fred Hutchinson Cancer Research Center, Seattle, WA 98109-1024 USA; 51 Department of Epidemiology, School of Public Health, University of Washington, Seattle, WA 98195 USA; 52 International Hereditary Cancer Center, Department of Genetics and Pathology, Pomeranian Medical University, 70-115 Szczecin, Poland; 53 Faculty of Health and Medical Sciences, University of Copenhagen, 2200 Copenhagen, Denmark; 54 Department of Clinical Biochemistry, Herlev and Gentofte Hospital, Copenhagen University Hospital, Herlev, 2200 Copenhagen, Denmark; 55 Division of Clinical Epidemiology and Aging Research, German Cancer Research Center (DKFZ), D-69120 Heidelberg, Germany; 56 German Cancer Consortium (DKTK), German Cancer Research Center (DKFZ), D-69120 Heidelberg, Germany; 57 Division of Preventive Oncology, German Cancer Research Center (DKFZ) and National Center for Tumor Diseases (NCT), 69120 Heidelberg, Germany; 58 Institute for Human Genetics, University Hospital Ulm, 89075 Ulm, Germany; 59 Department of Genitourinary Medical Oncology, The University of Texas MD Anderson Cancer Center, Houston, TX 77030 USA; 60 Department of Genetics, Portuguese Oncology Institute of Porto, 4200-072 Porto, Portugal; 61 Biomedical Sciences Institute (ICBAS), University of Porto, 4050-313 Porto, Portugal; 62 Department of Population Sciences, Beckman Research Institute of the City of Hope, Duarte, CA 91010 USA; 63 Ghent University, Faculty of Medicine and Health Sciences, Basic Medical Sciences, B-9000 Gent, Belgium; 64 Department of Surgery, Faculty of Medicine, University of Malaya, 50603 Kuala Lumpur, Malaysia; 65 Department of Urology, University of Washington, Seattle, WA 98195 USA; 66 Institute of Human Genetics, University Medical Center Hamburg-Eppendorf, D-20246 Hamburg, Germany; 67 Molecular Medicine Center, Department of Medical Chemistry and Biochemistry, Medical University of Sofia, 1431 Sofia, Bulgaria; 68 Department of Oncology, Cross Cancer Institute, University of Alberta, Edmonton, AB T6G 1Z2 Canada; 69 Division of Radiation Oncology, Cross Cancer Institute, University of Alberta, Edmonton, AB T6G 1Z2 Canada; 70 Molecular Endocrinology Laboratory, Department of Cellular and Molecular Medicine, KU Leuven, BE-3000 Leuven, Belgium; 71 Manchester Cancer Research Centre, Faculty of Biology Medicine and Health, Manchester Academic Health Science Centre, NIHR Manchester Biomedical Research Centre, Health Innovation Manchester, University of Manchester, Manchester, M13 9WL UK; 72 Genomic Medicine Group, Galician Foundation of Genomic Medicine, Instituto de Investigacion Sanitaria de Santiago de Compostela (IDIS), Complejo Hospitalario Universitario de Santiago, Servicio Galego de Saúde, SERGAS, 15706 Santiago de Compostela, Spain; 73 Moores Cancer Center, University of California San Diego, La Jolla, CA 92037 USA; 74 Department of Urology, Erasmus University Medical Center, 3015 CE Rotterdam, The Netherlands; 75 Cancer and Environment Group, Center for Research in Epidemiology and Population Health (CESP), INSERM, University Paris-Sud, University Paris-Saclay, 94807 Villejuif Cédex, France; 76 Clinical Gerontology Unit, University of Cambridge, Cambridge, CB2 2QQ UK; 77 Division of Genetic Epidemiology, Department of Medicine, University of Utah School of Medicine, Salt Lake City, UT 84112 USA; 78 George E. Wahlen Department of Veterans Affairs Medical Center, Salt Lake City, UT 84148 USA; 79 The University of Surrey, Guildford, Surrey, GU2 7XH UK; 80 Department of Laboratory Medicine and Pathology, Mayo Clinic, Rochester, MN 55905 USA; 81 Division of Biomedical Statistics and Informatics, Mayo Clinic, Rochester, MN 55905 USA; 82 Royal Marsden NHS Foundation Trust, London, SW3 6JJ UK; 83 Molecular Cancer Epidemiology Laboratory, QIMR Berghofer Institute of Medical Research, Herston, QLD 4006 Australia; 84 School of Medicine, University of Queensland, Herston, QLD 4006 Australia; 85 Royal Brisbane and Women’s Hospital, Herston, QLD 4029 Australia; 86 The Kinghorn Cancer Centre (TKCC), Victoria, NSW 2010 Australia; 87 Prostate Cancer Research Group, South Australian Health and Medical Research Institute, Adelaide, SA 5000 Australia; 88 Department of Physiology, Biomedicine Discovery Institute, Cancer Program, Monash University, Melbourne, VIC 3800 Australia; 89 University of Adelaide, North Terrace, Adelaide, SA 5005 Australia; 90 Fimlab Laboratories, Tampere University Hospital, FI-33520 Tampere, Finland; 91 Finnish Cancer Registry, FI-00130 Helsinki, Finland; 92 Department of Urology, Helsinki University Central Hospital and University of Helsinki, FI-00014 Helsinki, Finland; 93 Division of Biostatistics and Bioinformatics, University of Maryland Greenebaum Cancer Center, and Department of Epidemiology and Public Health, University of Maryland School of Medicine, Baltimore, MD 21201 USA; 94 Cancer Genomics Research Laboratory (CGR), Division of Cancer Epidemiology and Genetics, FNLCR Leidos Biomedical Research, National Cancer Institute, Frederick, MD 21701 USA; 95 DNA Extraction and Staging Laboratory (DESL), Cancer Genomics Research Laboratory (CGR), Division of Cancer Epidemiology and Genetics, FNLCR Leidos Biomedical Research, National Cancer Institute, Frederick, MD 21701 USA; 96 Sheffield Institute for Nucleic Acids, University of Sheffield, Sheffield, S10 2TN UK; 97 Cambridge Cancer Trials Centre, Cambridge Clinical Trials Unit-Cancer Theme, Cambridge University Hospitals NHS Foundation Trust, Cambridge, CB2 0QQ UK; 98 Department of Medical Imaging, University Health Network, Toronto, ON M5G 2C4 Canada; 99 Department of Pathology, University Health Network, Toronto, ON M5G 2C4 Canada; 100 Advanced Radiation Biology Research Program, Research Center for Charged Particle Therapy, National Institute of Radiological Sciences, Chiba, 263-8555 Japan; 101 Department of Urology, National Hospital Organization Tokyo Medical Center, Tokyo, 152-8902 Japan; 102 Department of Urology, Nanfang Hospital, Southern Medical University, 510515 Guangzhou, China; 103 Department of Pathology, The First Affiliated Hospital, Zhejiang University Medical College, 310009 Hangzhou, China; 104 Department of Pathology, Changhai Hospital, The Second Military Medical University, 200433 Shanghai, China; 105 Department of Urology, First Affiliated Hospital, Medical College, Zhengzhou University, 450003 Zhengzhou, China; 106 Department of Urology, North Sichuan Medical College, 637000 Nanchong, China; 107 Department of Nutrition Science, Shenyang Medical College, 110034 Shenyang, China; 108 Tissupath Pty Ltd., Melbourne, VIC, 3122 Australia; 109 Department of Medical Physics, Complexo Hospitalario Universitario de Santiago, SERGAS, 15706 Santiago de Compostela, Spain; 110 Urology Department, Hospital Germans Trias I Pujol, 08916 Barcelona, Spain; 111 Laboratory and Department of Urology, Hospital Clínic, Institut d’Investigacions Biomèdiques August Pi i Sunyer (IDIBAPS), Universitat de Barcelona, 08036 Barcelona, Spain; 112 Centre de Recerca Biomèdica CELLEX, 08036 Barcelona, Spain; 113 Department and Laboratory of Urology, Hospital Clínic, Institut d’Investigacions Biomèdiques August Pi i Sunyer (IDIBAPS), Universitat de Barcelona, 08036 Barcelona, Spain; 114 Genitourinary Program, Moffitt Cancer Center, Tampa, FL 33612 USA; 115 Department of Urology, Klinikum rechts der Isar der Technischen Universitaet Muenchen, 81675 Munich, Germany; 116 Department of General and Clinical Pathology, Alexandrovska University Hospital, Medical University, 1431 Sofia, Bulgaria; 117 Center of Excellence in Genomic Medicine Research, King Abdulaziz University, Jeddah, 2252 3270 Saudi Arabia; 118 Grupo de Medicina Xenómica, CIBERER, CIMUS, Universidad de Santiago de Compostela, Avenida de Barcelona, 15782 Santiago de Compostela, Spain; 119 Hérault Cancer Registry, Montpellier cedex 5, 34298 Montpellier, France; 120 Urology Department, Clinique Beau Soleil, 34070 Montpellier, France; 121 INSERM U1147, 75013 Paris, France; 122 Department of Clinical Science, Intervention and Technology, Karolinska Institutet, SE-171 77 Stockholm, Sweden; 123 Swedish Agency for Health Technology Assessment and Assessment of Social Services, SE-102 33 Stockholm, Sweden; 124 Department of Surgical and Perioperative Sciences, Urology and Andrology, Umeå University, SE-901 85 Umeå, Sweden; 125 Department of Surgical Sciences, Uppsala University, SE-751 85 Uppsala, Sweden; 126 Department of Urology, Faculty of Medicine and Health, Örebro University, SE-701 82 Örebro, Sweden; 127 Medical Research Council (MRC) Integrative Epidemiology Unit, University of Bristol, Bristol, BS8 2BN UK; 128 National Institute for Health Research (NIHR) Biomedical Research Centre, University of Bristol, Bristol, BS8 1TH UK; 129 Department of Urology, Cancer Therapy and Research Center, University of Texas Health Science Center at San Antonio, San Antonio, TX 78229 USA; 130 Menzies Health Institute Queensland, Griffith University, Gold Coast, QLD 4222 Australia; 131 Cancer Council Queensland, Fortitude Valley, QLD 4006 Australia; 132 Chris O’Brien Lifehouse (COBLH), Camperdown, Sydney, NSW 2010 Australia; 133 Garvan Institute of Medical Research, Sydney, NSW 2010 Australia; 134 Dame Roma Mitchell Cancer Research Centre, University of Adelaide, Adelaide, SA 5005 Australia; 135 Department of Anatomy and Developmental Biology, Biomedicine Discovery Institute, Monash University, Melbourne, VIC 3800 Australia; 136 Prostate Cancer Translational Research Program, Cancer Research Division, Peter MacCallum Cancer Centre, Melbourne, VIC 3000 Australia; 137 Department of Molecular Medicine and Surgery, Karolinska Institutet, and Department of Urology, Karolinska University Hospital, 171 76 Stockholm, Sweden; 138 Department of Clinical Sciences at Danderyd Hospital, Karolinska Institutet, 182 88 Stockholm, Sweden; 139 Cancer Genome Project, Wellcome Trust Sanger Institute, Hinxton, Cambridge, CB10 1SA UK; 140 Department of Urology, Tampere University Hospital, University of Tampere, Kalevantie 4, FI-33014 Tampere, Finland; 141 Faculty of Medicine and Life Sciences, University of Tampere, FI-33014 Tampere, Finland; 142 Department of Epidemiology, School of Health Sciences, University of Tampere, FI-33014 Tampere, Finland; 143 University of Cambridge Department of Oncology, Oncology Centre, Cambridge University Hospitals NHS Foundation Trust, Cambridge, CB1 8RN UK; 144 Washington University School of Medicine, StLouis, MO 63110 USA; 145 Department of Urology, Aarhus University Hospital, 8200 Aarhus N, Denmark; 146 Department of Radiation Oncology, University of Rochester Medical Center, Rochester, NY 14620 USA; 147 Department of Pathology and Pediatrics, Albert Einstein College of Medicine, Bronx, NY 10461 USA; 148 Second Military Medical University, 200433 Shanghai, China; 149 Wuxi Second Hospital, Nanjing Medical University, 214003 Wuxi, Jiangzhu China; 150 Department of Urology, The First Affiliated Hospital, Chongqing Medical University, 200032 Chongqing, China; 151 The People’s Hospital of Liaoning Province and The People’s Hospital of China Medical University, 110001 Shenyang, China; 152 Department of Urology, Zhongshan Hospital, Fudan University Medical College, 200032 Shanghai, China; 153 Precision Medicine, School and Clinical Sciences at Monash Health, Monash University, Clayton, VIC 3168 Australia; 154 Menzies Institute for Medical Research, University of Tasmania, Hobart, TAS 7000 Australia; 155 Faculty of Medical Science, John Radcliffe Hospital, University of Oxford, Oxford, OX1 2JD UK; 156 Department of Radiation Oncology, Complexo Hospitalario Universitario de Santiago, SERGAS, 15706 Santiago de Compostela, Spain; 157 Division of Family Medicine, Department of Neurobiology, Care Science and Society, Karolinska Institutet, Huddinge, SE-171 77 Stockholm, Sweden; 158 Scandinavian Development Services, 182 33 Danderyd, Sweden; 159 University of Cantabria-IDIVAL, 39005 Santander, Spain; 160 School of Public Health, Louisiana State University Health Sciences Center, New Orleans, LA 70112 USA; 161 National Human Genome Research Institute, National Institutes of Health, Bethesda, MD 20892 USA; 162 Department of Urology, Herlev and Gentofte Hospital, Copenhagen University Hospital, Herlev, 2200 Copenhagen, Denmark; 163 Copenhagen Prostate Cancer Center, Department of Urology, Rigshospitalet, Copenhagen University Hospital, DK-2730 Herlev, Denmark; 164 Saarland Cancer Registry, 66119 Saarbrücken, Germany; 165 Department of Urology, University Hospital Ulm, 89075 Ulm, Germany; 166 Cancer Prevention Institute of California, Fremont, CA 94538 USA; 167 Department of Health Research and Policy (Epidemiology) and Stanford Cancer Institute, Stanford University School of Medicine, Stanford, CA 94305-5101 USA; 168 Department of Radiotherapy, Ghent University Hospital, B-9000 Gent, Belgium; 169 Cancer Research Malaysia (CRM), Outpatient Centre, Subang Jaya Medical Centre, 47500 Subang Jaya, Selangor Malaysia; 170 Urogenital Unit, Division of Medical Oncology, Department of Oncology, University Hospital Centre Zagreb, Šalata 2, 10000 Zagreb, Croatia; 171 Department of Urology, University Hospital Center Zagreb, University of Zagreb School of Medicine, Šalata 2, 10000 Zagreb, Croatia; 172 Department of Urology and Alexandrovska University Hospital, Medical University of Sofia, 1431 Sofia, Bulgaria; 173 Department of Urology, University Hospitals Leuven, BE-3000 Leuven, Belgium; 174 Southampton General Hospital, The University of Southampton, Southampton, SO16 6YD UK; 175 Genetic Oncology Unit, CHUVI Hospital, Instituto de Investigación Biomédica Galicia Sur (IISGS), Complexo Hospitalario Universitario de Vigo, 36204 Vigo (Pontevedra), Spain; 176 Moores Cancer Center, Department of Family Medicine and Public Health, University of California San Diego, La Jolla, CA 92093-0012 USA; 177 Department of Clinical Chemistry, Erasmus University Medical Center, 3015 CE Rotterdam, The Netherlands; 178 Program for Personalized Cancer Care, NorthShore University HealthSystem, Evanston, IL 60201 USA; 179 Department of Epidemiology, University of Washington, Seattle, WA 98195 USA; 180 Department of Epidemiology and Biostatistics, School of Public Health, Imperial College, London, SW7 2AZ UK; 181 Genomics England, Queen Mary University of London, Dawson Hall, Charterhouse Square, London, EC1M 6BQ UK; 182 Genomic Epidemiology Group, German Cancer Research Center (DKFZ), D-69120 Heidelberg, Germany; 183 Epidemiology Program, University of Hawaii Cancer Center, Honolulu, HI 96813 USA; 184 Dana-Farber Cancer Institute, Boston, MA 02215 USA; 185 Paris-Sud University, UMRS 1018, Cedex 94807 Villejuif, France

## Abstract

Chromosome 8q24 is a susceptibility locus for multiple cancers, including prostate cancer. Here we combine genetic data across the 8q24 susceptibility region from 71,535 prostate cancer cases and 52,935 controls of European ancestry to define the overall contribution of germline variation at 8q24 to prostate cancer risk. We identify 12 independent risk signals for prostate cancer (*p* < 4.28 × 10^−15^), including three risk variants that have yet to be reported. From a polygenic risk score (PRS) model, derived to assess the cumulative effect of risk variants at 8q24, men in the top 1% of the PRS have a 4-fold (95%CI = 3.62–4.40) greater risk compared to the population average. These 12 variants account for ~25% of what can be currently explained of the familial risk of prostate cancer by known genetic risk factors. These findings highlight the overwhelming contribution of germline variation at 8q24 on prostate cancer risk which has implications for population risk stratification.

## Abstract (web summary)

Chromosome 8q24 is known to be a major susceptibility region for prostate cancer risk. Here the authors analyze genetic data across the 8q24 region from 71,535 prostate cancer patients identifying 12 risk loci, three previously unreported, highlighting the contribution of germline variation at this locus.

## Introduction

Prostate cancer (PCa) is the most common cancer among men in the US, with 161,360 new cases and 26,730 related deaths estimated in 2017^1^. Familial and epidemiological studies have provided evidence of substantial heritability of PCa^2^, and ~170 common risk loci have been identified through genome-wide association studies (GWAS)^3^. The susceptibility region on chromosome 8q24 has been shown to be a major contributor to PCa risk, with multiple variants clustered in five linkage disequilibrium (LD) blocks spanning ~600 Mb that are independently associated with risk^4^. Many of these association signals reported at 8q24 have been replicated across racial/ethnic populations^5,6^, pointing to common shared functional variants within 8q24. However, rare ancestry-specific variants have also been detected, which confer larger relative risks of PCa (odds ratios [ORs] >2.0) than common risk variants in the region and signify allelic heterogeneity in the contribution of germline variation at 8q24 to PCa risk across populations^7^.

In the current study, we perform a comprehensive investigation of genetic variation across the 1.4 Mb cancer susceptibility region at 8q24 (127.6–129.0 Mb) in relation to PCa risk. We combine genotyped and imputed data from two large GWAS consortia (PRACTICAL/ELLIPSE OncoArray and iCOGS) including >124,000 individuals of European ancestry to search for novel risk variants, as well as to determine the overall contribution of genetic variation at 8q24 to PCa heritability. Our findings underscore the sizable impact of genetic variation in the 8q24 region in explaining inter-individual differences in PCa risk, with potential clinical utility for genetic risk prediction.

## Results

### Marginal and conditional association analysis

**Fig. 1:**
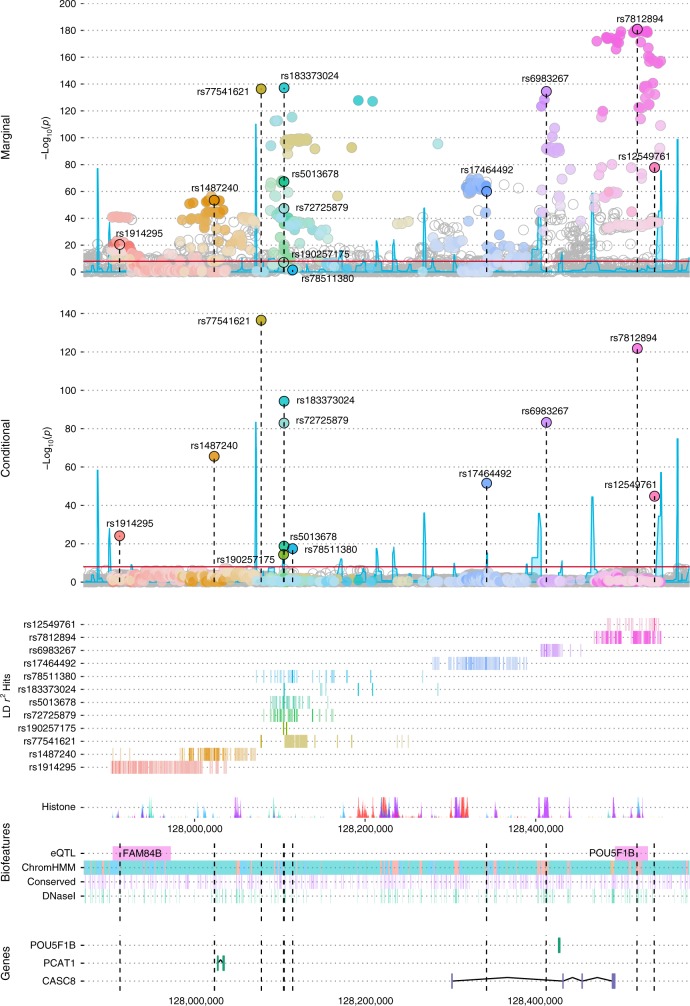
LocusExplorer plots of the 12 variants at 8q24 significantly associated with PCa risk. ‘Marginal’ and ‘Conditional’ Manhattan plot panels show marginal and conditional association results, respectively. Variant positions (*x*-axis) and −log_10_ *p-*values from the Wald test (*y*-axis) are shown, with the red line indicating the threshold for genome-wide significant association with PCa risk (*p* ≤ 5 × 10^−8^) and blue peaks local estimates of recombination rates. The position of the 12 independent variants is labeled in each plot. Clusters of correlated variants for each independent signal are distinguished using different colors and also depicted on the ‘LD *r*^2^ Hits’ track. Stronger shading indicates greater correlation with the lead variant, with variants not correlated at *r*^2^ ≥ 0.2 with any lead variant uncolored. Pairwise correlations are based on the European ancestry (EUR) panel from the 1000 Genomes Project (1KGP) Phase 3. The relative position of RefSeq genes and biological annotations are shown in the ‘Genes’ and ‘Biofeatures’ panels, respectively. Genes on the positive strand are denoted in green and those on the negative strand in purple. Annotations displayed are: histone modifications in ENCODE tier 1 cell lines (Histone track), the positions of any variants that were eQTLs with prostate tumor expression in TCGA prostate adenocarcinoma samples and the respective genes for which expression is altered (eQTL track), chromatin state categorizations in the PrEC cell-line by ChromHMM (ChromHMM track), the position of conserved element peaks (Conserved track) and the position of DNaseI hypersensitivity site peaks in ENCODE prostate cell-lines (DNaseI track). The data displayed in this plot may be explored interactively through the LocusExplorer application (http://www.oncogenetics.icr.ac.uk/8q24/)

Genotype data from the Illumina OncoArray and iCOGS array and imputation to 1000 Genomes Project (1KGP) were generated among 71,535 PCa cases and 52,935 controls of European ancestry from 86 case-control studies (see Methods). Of the 5600 genotyped and imputed variants at 8q24 (127.6–129.0 Mb) with minor allele frequency (MAF) > 0.1% retained for analysis (see Methods), 1268 (23%) were associated with PCa risk at *p* < 5×10^−8^ while 2772 (49%) were marginally associated at *p* < 0.05. These 5600 markers capture, at *r*^2^ > 0.8, 90% and 97% of all variants at 8q24 (127.6–129.0 Mb) with MAF ≥ 1% and ≥5%, respectively (based on 1KGP Phase 3 EUR panel). In a forward and backward stepwise selection model on variants marginally associated with PCa risk (*p* < 0.05, *n* = 2772; see Methods), we identified 12 variants with conditional *p*-values from the Wald test between 2.93 × 10^−137^ and 4.28 × 10^−15^ (Table 1). None of the other variants were statistically significant at *p* < 5 × 10^−8^ after adjustment for the 12 independent hits (Fig. 1). The 8q24 region is shown in Supplementary Fig. 1. Of these 12 stepwise signals, three had alleles with extreme risk allele frequencies (RAFs) that conveyed large effects (rs77541621, RAF = 2%, OR = 1.85, 95%CI = 1.76–1.94; rs183373024, RAF = 1%, OR = 2.67, 95%CI = 2.43–2.93; rs190257175, RAF = 99%, OR = 1.60, 95%CI = 1.42–1.80). The remaining variants had RAFs between 0.11 and 0.92 and conditional ORs that were more modest and ranged from 1.10 to 1.37 (Table 1). For 8 of the 12 variants, the allele found to be positively associated with PCa risk was the predominant allele (i.e., >50% in frequency). For two variants, rs78511380 and rs190257175, the marginal associations were not genome-wide significant and substantially weaker than those in the conditional model. For rs78511380, the marginal OR was slightly protective (OR = 0.97; *p* = 0.027), but reversed direction and was highly statistically significant when conditioning on the other 11 variants (OR = 1.19; *p* = 3.5 × 10^−18^; Table 1).

**Table 1:** Marginal and conditional estimates for genetic markers at 8q24 independently associated with prostate cancer risk

Variant ID^a^	Position^b^	Allele^c^	RAF^d^	LD cluster^e^	Conditional association^f^		Marginal association	
					OR (95%CI)^g^	*p*-value	OR (95%CI)^h^	*p*-value
rs1914295	127910317	T/C	0.68	block 1	1.10 (1.08–1.12)	7.30 × 10^−25^	1.09 (1.07–1.11)	3.07 × 10^−21^
rs1487240	128021752	A/G	0.74	block 1	1.20 (1.17–1.22)	2.77 × 10^−66^	1.16 (1.14–1.18)	2.97 × 10^−54^
rs77541621	128077146	A/G	0.02	block 2	1.85 (1.76–1.94)	2.93 × 10^−137^	1.83 (1.74–1.92)	4.33 × 10^−137^
rs190257175	128103466	T/C	0.99	block 2	1.60 (1.42–1.80)	4.28 × 10^−15^	1.36 (1.22–1.53)	6.90 × 10^−8^
rs72725879	128103969	T/C	0.18	block 2	1.31 (1.28–1.35)	1.26 × 10^−83^	1.17 (1.14–1.19)	3.96 × 10^−48^
rs5013678	128103979	T/C	0.78	block 2	1.10 (1.08–1.13)	1.58 × 10^−19^	1.20 (1.17–1.22)	4.44 × 10^−68^
rs183373024	128104117	G/A	0.01	block 2	2.67 (2.43–2.93)	4.89 × 10^−95^	3.20 (2.92–3.50)	6.60 × 10^−138^
rs78511380	128114146	T/A	0.92	block 2	1.19 (1.14–1.23)	3.48 × 10^−18^	0.97 (0.94–1.00)	0.027
rs17464492	128342866	A/G	0.72	block 3	1.16 (1.14–1.18)	3.01 × 10^−52^	1.17 (1.15–1.19)	9.05 × 10^−61^
rs6983267	128413305	G/T	0.51	block 4	1.18 (1.16–1.20)	5.68 × 10^−84^	1.23 (1.21–1.25)	3.15 × 10^−135^
rs7812894	128520479	A/T	0.11	block 5	1.37 (1.33–1.40)	1.55 × 10^−122^	1.45 (1.41–1.49)	1.20 × 10^−181^
rs12549761	128540776	C/G	0.87	block 5	1.21 (1.18–1.24)	1.61 × 10^−45^	1.28 (1.25–1.31)	1.38 × 10^−78^

^a^Variants that remained genome-wide significantly associated with PCa risk (*p* < 10^−8^) in the final stepwise model

^b^Chromosome position based on human genome build 37

^c^Risk allele/reference allele

^d^Risk allele frequency

^e^LD clusters were inferred based on recombination hotspots using Haploview 4.2^29^ and defined as previously reported by Al Olama et al.^4^

^f^Each variant was incorporated in the stepwise model based on the strength of marginal association from the meta-analysis of OncoArray and iCOGS data

^g^Per-allele odds ratio and 95% confidence interval adjusted for country, 7(OncoArray)/8(iCOGS) principal components and all other variants in the table

^h^Per-allele odds ratio and 95% confidence interval adjusted for country and 7(OncoArray)/8(iCOGS) principal components

### Haplotype analysis

The haplotype analysis showed an additive effect of the 12 independent risk variants consistent with that predicted in the single variant test; co-occurrence of the 8q24 risk alleles on the same haplotype does not further increase the risk of PCa (Supplementary Table 1). The unique haplotype carrying the reference allele for rs190257175 (GCTTAT, 0.5% frequency) is also the sole haplotype associated with a reduced risk of PCa, suggesting that having the C allele confers a protective effect. The reference allele for rs78511380 (A, 8% frequency) occurs on a haplotype in block 2 together with the risk alleles for rs190257175, rs72725879 and rs5013678 (haplotype GTTTAA, 8%) which obscures the positive association with the T allele of rs78511380. Thus, the marginal protective effect associated with the risk allele for rs78511380 reflects an increased risk associated with the occurrence on a risk haplotype with other risk alleles (Supplementary Table 1).

### Correlation with known risk loci

The 12 risk variants spanned across the five LD blocks previously reported to harbor risk variants for PCa at 8q24^4^, with block 2 harboring six signals, blocks 1 and 5 two signals each, and blocks 3 and 4 only one (Supplementary Fig. 2). Except for a weak correlation between rs72725879 and rs78511380 in block 2 (*r*^2^ = 0.28), the risk variants were uncorrelated with each other (*r*^2^ ≤ 0.09; Supplementary Data 1), which corroborates their independent association with PCa risk. Eight of the variants (rs1487240, rs77541621, rs72725879, rs5013678, rs183373024, rs17464492, rs6983267, rs7812894) have been previously reported either directly (Supplementary Table 2) or are correlated (*r*^2^ ≥ 0.42) with known markers of PCa risk from studies in populations of European, African or Asian ancestry (Supplementary Data 1)^4,7–10^. The marginal estimates for previously published PCa risk variants at 8q24 in the current study are shown in Supplementary Table 2. The variant rs1914295 in block 1 is only weakly correlated with the previously reported risk variants rs12543663 and rs10086908 (*r*^2^ = 0.17 and 0.14, respectively), while rs7851380 is modestly correlated with the previously reported risk variant rs1016343 (*r*^2^ = 0.28). The remaining two variants, rs190257175 and rs12549761, are not correlated (*r*^2^ < 0.027) with any known PCa risk marker.

### Polygenic risk score and familial relative risk

To estimate the cumulative effect of germline variation at 8q24 on PCa risk, a polygenic risk score (PRS) was calculated for the 12 independent risk alleles from the final model based on allele dosages weighted by the per-allele conditionally adjusted ORs (see Methods). Compared to the men at ‘average risk’ (i.e., the 25th–75th PRS range among controls), men in the top 10% of the PRS distribution had a 1.93-fold relative risk (95%CI = 1.86–2.01) (Table 2), with the risk being 3.99-fold higher (95%CI = 3.62–4.40) for men in the top 1%. Risk estimates by PRS category were not modified by family history (FamHist-yes: OR = 4.24, 95%CI = 2.85–6.31; FamHist-no: OR = 3.38, 95%CI = 2.88–3.97). To quantify the impact of germline variation at 8q24, we also estimated the proportion of familial relative risk (FRR) and heritability of PCa contributed by 8q24 and compared this to the proportions explained by all known PCa risk variants including 8q24 (see Methods). The 175 established PCa susceptibility loci identified to date^3,11^ are estimated to explain 37.08% (95%CI = 32.89–42.49) of the FRR of PCa, while the 12 independent signals at 8q24 alone capture 9.42% (95%CI = 8.22–10.88), which is 25.4% of the total FRR explained by known genetic risk factors for PCa (Table 3). This is similar to the proportion of heritability explained by 8q24 variants (22.2%) compared to the total explained heritability by the known risk variants (0.118). In comparison, the next highest contribution of an individual susceptibility region to the FRR of PCa is the *TERT* region at chromosome 5p15, where 5 independent signals contributed 2.63% (95%CI = 2.34–3.00). No other individual GWAS locus has been established as explaining >2% of the FRR, including the low frequency, non-synonymous, moderate penetrance *HOXB13* variant (rs138213197) at chromosome 17q21 that is estimated to explain only 1.91% (95%CI = 1.20–2.85) of the FRR^11^.

**Table 2:** Relative risk of PCa for polygenic risk score (PRS) groups

Risk category percentile^a^	No. of individuals		Risk estimates for PRS groups	
	Controls	Cases	OR (95% CI)^b^	*p*-value
≤1%	530	339	0.52 (0.45–0.59)	2.11 × 10^−20^
1%–10%	4771	3636	0.62 (0.59–0.65)	6.26 × 10^−90^
10%–25%	7936	7359	0.75 (0.72–0.78)	3.62 × 10^−54^
25%–75%	26,464	32,743	1.00 (Ref)	
75%–90%	7940	13,431	1.37 (1.32–1.41)	6.55 × 10^−77^
90%–99%	4766	11,451	1.93 (1.86–2.01)	4.13 × 10^−249^
>99%	528	2576	3.99 (3.62–4.40)	5.64 × 10^−172^

*Note*: PRS were calculated for variants from the final stepwise model with allele dosage from OncoArray and iCOGS weighted by the per-allele conditionally adjusted odds ratios from the meta-analysis

^a^Risk category groups were based on the percentile distribution of risk alleles in overall controls

^b^Estimated effect of each PRS group relative to the interquartile range (25–75%) in OncoArray and iCOGS datasets separately, and then meta-analyzed across the two studies; odds ratios were adjusted for country and 7(OncoArray)/8(iCOGS) principal components

**Table 3:** Proportion of familial relative risk (FRR) and heritability (h_g_^2^) of PCa explained by known risk variants

Source	No. of variants	Proportion of FRR (95%CI)	% of total FRR	h_g_^2^ (SE)	% of total h_g_^2^
8q24^a^	12	9.42 (8.22–10.88)	25.4	0.027 (0.011)	22.2
HOXB13^b^	1	1.91 (1.20–2.85)	5.2	0.004 (0.005)	3.0
All other variants^b,c^	162	25.77 (22.94–29.36)	69.5	0.092 (0.010)	74.9
Total	175	37.08 (32.89–42.49)	100	0.118 (0.012)	100

^a^Conditional estimates were derived by fitting a single model with all variants from OncoArray data

^b^Risk estimates and allele frequencies for regions with a single variant are from a meta-analysis of OncoArray, iCOGS and 6 additional GWAS^3^

^c^Risk variants included from fine-mapping of PCa susceptibility loci in European ancestry populations^11^

### JAM analysis

We explored our data with a second fine-mapping approach, JAM (Joint Analysis of Marginal summary statistics)^12^, which uses GWAS summary statistics to identify credible sets of variants that define the independent association signals in susceptibility regions (see Methods). The 95% credible set for the JAM analysis confirmed all of the independent signals from stepwise analysis except rs190257175, for which evidence for an association was weak (variant-specific Bayes factor (BF) = 1.17). There were 50 total variants included in the 95% credible set, and 174 after including variants in high LD (*r*^2^ > 0.9) with those in the credible set (Supplementary Data 2).

## Discussion

In this large study of germline genetic variation across the 8q24 region, we identified 12 independent association signals among men of European ancestry, with three of the risk variants (rs1914295, rs190257175, and rs12549761) being weakly correlated (*r*^2^ ≤ 0.17) with known PCa risk markers. The combination of these 12 independent signals at 8q24 capture approximately one quarter of the total PCa FRR explained by known genetic risk factors, which is substantially greater than any other known PCa risk locus.

The 8q24 region is the major susceptibility region for PCa; however, the underlying biological mechanism(s) through which germline variation in this region influences PCa risk remains uncertain. For each of the 12 risk variants at 8q24, the 95% credible set defined noteworthy (i.e., putative functional) variants based on summary statistics while accounting for LD. To inform biological functionality, we overlaid epigenetic functional annotation using publicly available datasets (see Methods) with the location of the 12 independent signals (and corresponding 174 variants within their 95% credible sets; Supplementary Data 3). Of the 12 independent lead variants, 6 are situated within putative transcriptional enhancers in prostate cell-lines; either through intersection with H3K27AC (rs72725879, rs5013678, rs78511380, rs6983267 and rs7812894) or through a ChromHMM enhancer annotation (rs17464492, rs6983267, rs7812894). Eight of the 12 stepwise hits (rs77541621, rs190257175, rs5013678, rs183373024, rs78511380, rs17464492, rs6983267, rs7812894) also intersect transcription factor binding site peaks from multiple ChIP-seq datasets representing the AR, ERG, FOXA1, GABPA, GATA2, HOXB13, and NKX3.1 transcription factors, with all 8 intersecting a FOXA1 mark and half an AR binding site. These variants may therefore exert their effect through regulation of enhancer activity and long-range expression of genes important for cancer tumorigenesis and/or progression^13^. The variant rs6983267 has also been shown to act in an allele-specific manner to regulate prostate enhancer activity and expression of the proto-oncogene *MYC* in vitro and in vivo^14,15^. However, despite the close proximity to the *MYC* locus, no direct association has been detected between 8q24 risk alleles and *MYC* expression in normal and tumor human prostate tissues^16^. The rare variant with the largest effect on risk, rs183373024, shows high evidence of functionality based on overlap with multiple DNaseI and transcription factor binding site peaks (for AR, FOXA1, HOXB13, and NKX3.1), which supports previous findings of an allele-dependent effect of this variant on the disruption of a FOXA1 binding motif^17^. Seven independent signals (rs1914295, rs1487240, rs77541621, rs72725879, rs5013678, rs183373024, rs78511380) and variants correlated at *r*^2^ > 0.9 with these signals (Supplementary Data 2) are located within or near a number of prostate cancer–associated long noncoding RNAs (lncRNAs), including *PRNCR1*, *PCAT1*, and *CCAT2*, previously reported to be upregulated in human PCa cells^18^ and tissues^19,20^. Based on eQTL annotations in prostate adenocarcinoma cells, the independent signal rs1914295 and three correlated variants (*r*^2^ > 0.9; Supplementary Data 2) are associated with overexpression of *FAM84B*, a gene previously associated with progression and poor prognosis of PCa in animal studies^21^. Variants correlated at *r*^2^ > 0.9 with rs7812894 (*n* = 9; Supplemental Table 4) are eQTLs for *POU5F1B*, a gene overexpressed in cancer cell lines and cancer tissues^22,23^, although its role in PCa development is unknown. Whilst we have successfully refined the 8q24 region and identified a subset of variants with putative biological function within our credible set, multi-ethnic comparisons may help refine the association signals even further and precisely identify the functional alleles and biological mechanisms that modify PCa risk.

Whereas the individual associations of the 8q24 variants with PCa risk are relatively modest (ORs < 2.0, except for rs183373024), their cumulative effects are substantial, with risk being 4-fold higher for men in the top 1% of the 8q24-only PRS. The contribution to the overall FRR of PCa is substantially greater for the 8q24 region (9.42%) than for any other known GWAS locus, including the moderate penetrance non-synonymous variant in *HOXB13* (1.91%). The ability of these markers to explain ~25.4% of what can be currently explained by all known PCa risk variants is a clear indication of the important contribution of germline variation at 8q24 on PCa risk. Our study was predominantly powered to analyze variants with MAF > 1% as the imputed variants with MAF = 0.1-1% were most likely to fail quality control (QC); however, the high density of genotyped markers and haplotypes at 8q24 in the OncoArray and iCOGS studies provided a robust backbone for imputation and increased the chances to impute lower MAF variants with high imputation quality score. Understanding of the biology of these variants and the underlying genetic basis of PCa could provide new insights into the identification of reliable risk-prediction biomarkers for PCa, as well as enable the development of effective strategies for targeted screening and prevention.

## Methods

### Study subjects, genotyping, and quality control

We combined genotype data from the PRACTICAL/ELLIPSE OncoArray and iCOGS consortia^3,24^, which included 143,699 men of European ancestry from 86 case-control studies largely based in either the US or Europe. In each study, cases primarily included men with incident PCa while controls were men without a prior diagnosis of the disease.

Both of the OncoArray and iCOGS custom arrays were designed to provide high coverage of common alleles (minor allele frequency [MAF] > 5%) across 8q24 (127.6–129.0 Mb) based on the 1000 Genomes Project (1KGP) Phase 3 for OncoArray, and the European ancestry (EUR) panel from HapMap Phase 2 for iCOGS. A total of 57,580 PCa cases and 37,927 controls of European ancestry were genotyped with the Illumina OncoArray, and 24,198 PCa cases and 23,994 controls of European ancestry were genotyped with the Illumina iCOGS array. For both studies, sample exclusion criteria included duplicate samples, first-degree relatives, samples with a call rate <95% or with extreme heterozygosity (*p* < 10^−6^), and samples with an estimated proportion of European ancestry <0.8^3,24^. In total, genotype data for 53,449 PCa cases and 36,224 controls from OncoArray and 18,086 PCa cases and 16,711 controls from iCOGS were included in the analysis. Genetic variants with call rates <0.95, deviation from Hardy-Weinberg equilibrium (*p* < 10^−7^ in controls), and genotype discrepancy in >2% of duplicate samples were excluded. Of the final 498,417 genotyped variants on the OncoArray and 201,598 on the iCOGS array that passed QC, 1581 and 1737 within the 8q24 region, respectively, were retained for imputation.

All studies complied with all relevant ethical regulations and were approved by the institutional review boards at each of the participating institutions. Informed consent was obtained from all study participants. Additional details of each study are provided in the Supplementary Note 1.

### Imputation analysis

Imputation of both OncoArray and iCOGS genotype data was performed using SHAPEIT^25^ and IMPUTEv2^26^ to the October 2014 (Phase 3) release of the 1KGP reference panel. A total of 10,136 variants from OncoArray and 10,360 variants from iCOGS with MAF > 0.1% were imputed across the risk region at 8q24 (127.6-129.0 Mb). Variants with an imputation quality score >0.8 were retained for a total of 5600 overlapping variants between the two datasets.

### Statistical analysis

Unconditional logistic regression was used to estimate per-allele odds ratios (ORs) and 95% confidence intervals (CIs) for the association between genetic variants (single nucleotide polymorphisms and insertion/deletion polymorphisms) and PCa risk adjusting for country and principal components (7 for OncoArray and 8 for iCOGS). Allele dosage effects were tested through a 1-degree of freedom two-tailed Wald trend test. The marginal risk estimates for the 5600 variants at 8q24 that passed QC were combined by a fixed effect meta-analysis with inverse variance weighting using METAL^27^. A modified forward and backward stepwise model selection with inclusion and exclusion criteria of *p* ≤ 5 × 10^−8^ was performed on variants marginally associated with PCa risk from the meta results (*p* < 0.05, *n* = 2772). At each step, the effect estimates for the candidate variants from both studies (OncoArray and iCOGS) were meta-analyzed and each variant was incorporated into the model based on the strength of association. All remaining variants were included one-at-a-time into the logistic regression model conditioning on those already incorporated in the model. We applied a conservative threshold for independent associations, with variants kept in the model if their meta p-value from the Wald test was genome-wide significant at *p* ≤ 5 × 10^−8^ after adjustment for the other variants in the model. Correlations between variants in the final model and previously published PCa risk variants at 8q24 were estimated using the 1KGP Phase 3 EUR panel (Supplementary Data 1).

### Haplotype analysis

Haplotypes were estimated in the Oncoarray data only using variants from the final stepwise model selection (*n* = 12) and the EM algorithm^28^ within LD block regions inferred based on recombination hotspots using Haploview 4.2 (Broad Institute, Cambridge, MA, USA)^29^. Only haplotypes with an estimated frequency ≥0.5% were tested.

### Polygenic risk score and familial relative risk

An 8q24-only polygenic risk score (PRS) was calculated for variants from the final model (*n* = 12) with allele dosage from OncoArray and iCOGS weighted by the per-allele conditionally adjusted ORs from the meta-analysis. Categorization of the PRS was based on the percentile distribution in controls, and the risk for each category was estimated relative to the interquartile range (25–75%) in OncoArray and iCOGS separately, and then meta-analyzed across the two studies. We estimated the contribution of 8q24 variants to the familial (first-degree) relative risk (FRR) of PCa (FRR = 2.5)^30^ under a multiplicative model, and compared this to the FRR explained by all known PCa risk variants including 8q24 (Supplementary Data 4). We also estimated heritability of PCa using the LMM approach as implemented in GCTA^31^. For regions which have been fine-mapped using the OncoArray meta-analysis data, we used the updated representative lead variants, otherwise the originally reported variant was included provided that it had replicated at genome-wide significance in the meta-analysis; this identified a total of 175 independently associated PCa variants for the FRR and heritability calculations^3,11^. For these analyses, we used conditional estimates from fitting a single model with all variants in the OncoArray dataset for regions with multiple variants and the overall marginal meta-analysis results from Schumacher et al.^3^ for regions with a single variant. To correct for potential bias in effect estimation of newly discovered variants, we implemented a Bayesian version of the weighted correction^32^, which incorporates the uncertainty in the effect estimate into the final estimates of the bias-corrected ORs, 95%CIs and the corresponding calculations of percent FRR explained.

### JAM analysis

To confirm the stepwise results and identify candidate variants for potential functional follow-up, we used a second fine-mapping approach, JAM (Joint Analysis of Marginal summary statistics)^12^. JAM is a multivariate Bayesian variable selection framework that uses GWAS summary statistics to identify the most likely number of independent associations within a locus and define credible sets of variants driving those associations. JAM was applied to summary statistics from the meta-analysis results using LD estimated from imputed individual level data from 20,000 cases and 20,000 controls randomly selected from the OncoArray sub-study. LD pruning was performed using Priority Pruner (http://prioritypruner.sourceforge.net/) on the 2772 marginally associated variants at *r*^2^ = 0.9, resulting in 825 tag variants analyzed in four independent JAM runs with varying starting seeds. Credible sets were determined as the tag variants that were selected in the top models that summed to a specific cumulative posterior probability in all four of the independent JAM runs, plus their designated high LD proxy variants from the pruning step.

### Functional annotation

Variants in the 95% credible set (*n* = 50) plus variants correlated at *r*^2^ > 0.9 with those in the credible set (*n* = 174) were annotated for putative evidence of biological functionality using publicly available datasets as described by Dadaev et al.^11^. Briefly, variants were annotated for proximity to gene (GENCODEv19), miRNA transcripts (miRBase release 20), evolutionary constraint (according to GERP++, SiPhy and PhastCons algorithms), likelihood of pathogenicity (CADDv1.3) and overlap with prospective regulatory elements in prostate-specific datasets (DNaseI hypersensitivity sites, H3K27Ac, H3K27me3 and H3K4me3 histone modifications, and for AR, CTCF, ERG, FOXA1, GABPA, GATA2, HOXB13, and NKX3.1 transcription factor binding sites) in a mixture of LNCaP, PC-3, PrEC, RWPE1, and VCaP cell lines and human prostate tumor tissues downloaded from the Cistrome Data Browser (http://cistrome.org/db/). The chromatin state in which each variant resides was assessed using ChromHMM annotations from two prostate cell lines (PrEC and PC3). Cis-gene regulation was evaluated using 359 prostate adenoma cases from The Cancer Genome Atlas (TCGA PRAD; https://gdc-portal.nci.nih.gov) that passed QC^11^. The eQTL analysis was performed using FastQTL with 1000 permutations for each gene within a 1Mb window. We then used the method by Nica et al.^33^ that integrates eQTLs and GWAS results in order to reveal the subset of association signals that are due to cis eQTLs. For each significant eQTL, we added the candidate variant to the linear regression model to assess if the inclusion better explains the change in expression of the gene. We retrieved the *p*-value of the model, assigning p-value of 1 if the eQTL and variant are the same. Then we ranked the p-values in descending order for each eQTL, and finally calculated the colocalization score for each pair of eQTL and variants. In general, if an eQTL and candidate variant represent the same signal, this will be reflected by the variant having a high p-value, a low rank and consequently a high colocalization score.

## Electronic supplementary material

Supplementary Information

Peer Review File

Supplementary Data 1

Supplementary Data 2

Supplementary Data 3

Supplementary Data 4

Description of Additional Supplementary Files

## Data Availability

The authors declare that data supporting the findings of this study are available within the paper [and in the supplementary information files]. However, some of the data used to generate the results of this study are available from the first author and the PRACTICAL Consortium upon request.

## References

[1] Siegel RL, Miller KD, Jemal A (2017). Cancer statistics, 2017. CA Cancer J. Clin..

[2] Hjelmborg JB (2014). The heritability of prostate cancer in the Nordic Twin Study of Cancer. Cancer Epidemiol. Prev. Biomark..

[3] Schumacher, F. R. et al. Association analyses of more than 140,000 men identify 63 new prostate cancer susceptibility loci. *Nat. Genet*. **50**, 928–936 (2018). 10.1038/s41588-018-0142-8 PMC6568012 29892016

[4] Al Olama AA (2009). Multiple loci on 8q24 associated with prostate cancer susceptibility. Nat. Genet..

[5] Haiman CA (2007). Multiple regions within 8q24 independently affect risk for prostate cancer. Nat. Genet..

[6] Han Y (2015). Generalizability of established prostate cancer risk variants in men of African ancestry. Int. J. Cancer.

[7] Gudmundsson J (2012). A study based on whole-genome sequencing yields a rare variant at 8q24 associated with prostate cancer. Nat. Genet..

[8] Han, Y. et al. Prostate cancer susceptibility in men of African ancestry at 8q24. *J. Natl Cancer Inst*. **108**, djv431 (2016). 10.1093/jnci/djv431 PMC4948565 26823525

[9] Hoffmann TJ (2015). A large multiethnic genome-wide association study of prostate cancer identifies novel risk variants and substantial ethnic differences. Cancer Discov..

[10] Conti, D. V. et al. Two novel susceptibility loci for prostate cancer in men of African ancestry. *J. Natl Cancer. Inst*. **109**, djx084 (2017). 10.1093/jnci/djx084 PMC5448553 29117387

[11] Dadaev T (2018). Fine-mapping of prostate cancer susceptibility loci in a large meta-analysis identifies candidate causal variants. Nat. Commun..

[12] Newcombe PJ, Conti DV, Richardson S (2016). JAM: a scalable Bayesian framework for joint analysis of marginal SNP effects. Genet. Epidemiol..

[13] Jia L (2009). Functional enhancers at the gene-poor 8q24 cancer-linked locus. PLoS Genet..

[14] Pomerantz MM (2009). The 8q24 cancer risk variant rs6983267 shows long-range interaction with MYC in colorectal cancer. Nat. Genet..

[15] Wasserman NF, Aneas I, Nobrega MA (2010). An 8q24 gene desert variant associated with prostate cancer risk confers differential in vivo activity to a MYC enhancer. Genome Res..

[16] Pomerantz MM (2009). Evaluation of the 8q24 prostate cancer risk locus and MYC expression. Cancer Res..

[17] Hazelett DJ, Coetzee SG, Coetzee GA (2013). A rare variant, which destroys a FoxA1 site at 8q24, is associated with prostate cancer risk. Cell Cycle Georget. Tex..

[18] Chung S (2011). Association of a novel long non-coding RNA in 8q24 with prostate cancer susceptibility. Cancer Sci..

[19] Prensner JR (2011). Transcriptome sequencing across a prostate cancer cohort identifies PCAT-1, an unannotated lincRNA implicated in disease progression. Nat. Biotechnol..

[20] Zheng J (2016). The up-regulation of long non-coding RNA CCAT2 indicates a poor prognosis for prostate cancer and promotes metastasis by affecting epithelial-mesenchymal transition. Biochem. Biophys. Res. Commun..

[21] Wong N (2017). Upregulation of FAM84B during prostate cancer progression. Oncotarget.

[22] Suo G (2005). Oct4 pseudogenes are transcribed in cancers. Biochem. Biophys. Res. Commun..

[23] Hayashi H (2015). The OCT4 pseudogene POU5F1B is amplified and promotes an aggressive phenotype in gastric cancer. Oncogene.

[24] Eeles RA (2013). Identification of 23 new prostate cancer susceptibility loci using the iCOGS custom genotyping array. Nat. Genet..

[25] Delaneau O, Marchini J, Zagury JF (2011). A linear complexity phasing method for thousands of genomes. Nat. Methods.

[26] Howie BN, Donnelly P, Marchini J (2009). A flexible and accurate genotype imputation method for the next generation of genome-wide association studies. PLoS Genet..

[27] Willer CJ, Li Y, Abecasis GR (2010). METAL: fast and efficient meta-analysis of genomewide association scans. Bioinforma. Oxf. Engl..

[28] Excoffier L, Slatkin M (1995). Maximum-likelihood estimation of molecular haplotype frequencies in a diploid population. Mol. Biol. Evol..

[29] Barrett JC, Fry B, Maller J, Daly MJ (2005). Haploview: analysis and visualization of LD and haplotype maps. Bioinforma. Oxf. Engl..

[30] Johns LE, Houlston RS (2003). A systematic review and meta-analysis of familial prostate cancer risk. BJU Int..

[31] Yang J, Lee SH, Goddard ME, Visscher PM (2011). GCTA: a tool for genome-wide complex trait analysis. Am. J. Hum. Genet..

[32] Zhong H, Prentice RL (2008). Bias-reduced estimators and confidence intervals for odds ratios in genome-wide association studies. Biostat. Oxf. Engl..

[33] Nica AC (2010). Candidate causal regulatory effects by integration of expression QTLs with complex trait genetic associations. PLoS Genet..

