# Author Correction: Germline variation at 8q24 and prostate cancer risk in men of European ancestry

**DOI:** 10.1038/s41467-019-08293-z

**Published:** 2019-01-17

**Authors:** Marco Matejcic, Edward J. Saunders, Tokhir Dadaev, Mark N. Brook, Kan Wang, Xin Sheng, Ali Amin Al Olama, Fredrick R. Schumacher, Sue A. Ingles, Koveela Govindasami, Sara Benlloch, Sonja I. Berndt, Demetrius Albanes, Stella Koutros, Kenneth Muir, Victoria L. Stevens, Susan M. Gapstur, Catherine M. Tangen, Jyotsna Batra, Judith Clements, Henrik Gronberg, Nora Pashayan, Johanna Schleutker, Alicja Wolk, Catharine West, Lorelei Mucci, Peter Kraft, Géraldine Cancel-Tassin, Karina D. Sorensen, Lovise Maehle, Eli M. Grindedal, Sara S. Strom, David E. Neal, Freddie C. Hamdy, Jenny L. Donovan, Ruth C. Travis, Robert J. Hamilton, Barry Rosenstein, Yong-Jie Lu, Graham G. Giles, Adam S. Kibel, Ana Vega, Jeanette T. Bensen, Manolis Kogevinas, Kathryn L. Penney, Jong Y. Park, Janet L. Stanford, Cezary Cybulski, Børge G. Nordestgaard, Hermann Brenner, Christiane Maier, Jeri Kim, Manuel R. Teixeira, Susan L. Neuhausen, Kim De Ruyck, Azad Razack, Lisa F. Newcomb, Davor Lessel, Radka Kaneva, Nawaid Usmani, Frank Claessens, Paul A. Townsend, Manuela Gago-Dominguez, Monique J. Roobol, Florence Menegaux, Kay-Tee Khaw, Lisa A. Cannon-Albright, Hardev Pandha, Stephen N. Thibodeau, Daniel J. Schaid, The PRACTICAL Consortium, Brian E. Henderson, Mariana C. Stern, Alison Thwaites, Michelle Guy, Ian Whitmore, Angela Morgan, Cyril Fisher, Steve Hazel, Naomi Livni, Margaret Cook, Laura Fachal, Stephanie Weinstein, Laura E. Beane Freeman, Robert N. Hoover, Mitchell J. Machiela, Artitaya Lophatananon, Brian D. Carter, Phyllis Goodman, Leire Moya, Srilakshmi Srinivasan, Mary-Anne Kedda, Trina Yeadon, Allison Eckert, Martin Eklund, Carin Cavalli-Bjoerkman, Alison M. Dunning, Csilla Sipeky, Niclas Hakansson, Rebecca Elliott, Hardeep Ranu, Edward Giovannucci, Constance Turman, David J. Hunter, Olivier Cussenot, Torben Falck Orntoft, Athene Lane, Sarah J. Lewis, Michael Davis, Tim J. Key, Paul Brown, Girish S. Kulkarni, Alexandre R. Zlotta, Neil E. Fleshner, Antonio Finelli, Xueying Mao, Jacek Marzec, Robert J. MacInnis, Roger Milne, John L. Hopper, Miguel Aguado, Mariona Bustamante, Gemma Castaño-Vinyals, Esther Gracia-Lavedan, Lluís Cecchini, Meir Stampfer, Jing Ma, Thomas A. Sellers, Milan S. Geybels, Hyun Park, Babu Zachariah, Suzanne Kolb, Dominika Wokolorczyk, Jan Lubinski, Wojciech Kluzniak, Sune F. Nielsen, Maren Weisher, Katarina Cuk, Walther Vogel, Manuel Luedeke, Christopher J. Logothetis, Paula Paulo, Marta Cardoso, Sofia Maia, Maria P. Silva, Linda Steele, Yuan Chun Ding, Gert De Meerleer, Sofie De Langhe, Hubert Thierens, Jasmine Lim, Meng H. Tan, Aik T. Ong, Daniel W. Lin, Darina Kachakova, Atanaska Mitkova, Vanio Mitev, Matthew Parliament, Guido Jenster, Christopher Bangma, F. H. Schroder, Thérèse Truong, Yves Akoli Koudou, Agnieszka Michael, Andrzej Kierzek, Ami Karlsson, Michael Broms, Huihai Wu, Claire Aukim-Hastie, Lori Tillmans, Shaun Riska, Shannon K. McDonnell, David Dearnaley, Amanda Spurdle, Robert Gardiner, Vanessa Hayes, Lisa Butler, Renea Taylor, Melissa Papargiris, Pamela Saunders, Paula Kujala, Kirsi Talala, Kimmo Taari, Søren Bentzen, Belynda Hicks, Aurelie Vogt, Amy Hutchinson, Angela Cox, Anne George, Ants Toi, Andrew Evans, Theodorus H. van der Kwast, Takashi Imai, Shiro Saito, Shan-Chao Zhao, Guoping Ren, Yangling Zhang, Yongwei Yu, Yudong Wu, Ji Wu, Bo Zhou, John Pedersen, Ramón Lobato-Busto, José Manuel Ruiz-Dominguez, Lourdes Mengual, Antonio Alcaraz, Julio Pow-Sang, Kathleen Herkommer, Aleksandrina Vlahova, Tihomir Dikov, Svetlana Christova, Angel Carracedo, Brigitte Tretarre, Xavier Rebillard, Claire Mulot, Jan Adolfsson, Par Stattin, Jan-Erik Johansson, Richard M. Martin, Ian M. Thompson, Suzanne Chambers, Joanne Aitken, Lisa Horvath, Anne-Maree Haynes, Wayne Tilley, Gail Risbridger, Markus Aly, Tobias Nordström, Paul Pharoah, Teuvo L. J Tammela, Teemu Murtola, Anssi Auvinen, Neil Burnet, Gill Barnett, Gerald Andriole, Aleksandra Klim, Bettina F. Drake, Michael Borre, Sarah Kerns, Harry Ostrer, Hong-Wei Zhang, Guangwen Cao, Ji Lin, Jin Ling, Meiling Li, Ninghan Feng, Jie Li, Weiyang He, Xin Guo, Zan Sun, Guomin Wang, Jianming Guo, Melissa C. Southey, Liesel M FitzGerald, Gemma Marsden, Antonio Gómez-Caamaño, Ana Carballo, Paula Peleteiro, Patricia Calvo, Robert Szulkin, Javier Llorca, Trinidad Dierssen-Sotos, Ines Gomez-Acebo, Hui-Yi Lin, Elaine A. Ostrander, Rasmus Bisbjerg, Peter Klarskov, Martin Andreas Røder, Peter Iversen, Bernd Holleczek, Christa Stegmaier, Thomas Schnoeller, Philipp Bohnert, Esther M. John, Piet Ost, Soo-Hwang Teo, Marija Gamulin, Tomislav Kulis, Zeljko Kastelan, Chavdar Slavov, Elenko Popov, Thomas Van den Broeck, Steven Joniau, Samantha Larkin, Jose Esteban Castelao, Maria Elena Martinez, Ron H. N. van Schaik, Jianfeng Xu, Sara Lindstrӧm, Elio Riboli, Clare Berry, Afshan Siddiq, Federico Canzian, Laurence N. Kolonel, Loic Le Marchand, Matthew Freedman, Sylvie Cenee, Marie Sanchez, Fredrik Wiklund, Stephen J. Chanock, Douglas F. Easton, Rosalind A. Eeles, Zsofia Kote-Jarai, David V. Conti, Christopher A. Haiman

**Affiliations:** 1 Department of Preventive Medicine, Keck School of Medicine, University of Southern California/Norris Comprehensive Cancer Center, Los Angeles, CA 90033 USA; 2 The Institute of Cancer Research, London, SW7 3RP UK; 3 Centre for Cancer Genetic Epidemiology, Department of Public Health and Primary Care, Strangeways Research Laboratory, University of Cambridge, Cambridge, CB1 8RN UK; 4 Department of Clinical Neurosciences, University of Cambridge, Cambridge, CB2 0QQ UK; 5 Department of Population and Quantitative Health Sciences, Case Western Reserve University, Cleveland, OH 44106-7219 USA; 6 Seidman Cancer Center, University Hospitals, Cleveland, OH 44106 USA; 7 Division of Cancer Epidemiology and Genetics, National Cancer Institute, NIH, Bethesda, MD 20892 USA; 8 Institute of Population Health, University of Manchester, Manchester, M13 9PL UK; 9 Warwick Medical School, University of Warwick, Coventry, CV4 7AL UK; 10 Epidemiology Research Program, American Cancer Society, 250 Williams Street, Atlanta, GA 30303 USA; 11 SWOG Statistical Center, Fred Hutchinson Cancer Research Center, Seattle, WA 98109 USA; 12 Australian Prostate Cancer Research Centre-Qld, Institute of Health and Biomedical Innovation and School of Biomedical Science, Queensland University of Technology, Brisbane, QLD 4059 Australia; 13 Translational Research Institute, Brisbane, QLD 4102 Australia; 14 Department of Medical Epidemiology and Biostatistics, Karolinska Institute, SE-171 77 Stockholm, Sweden; 15 Centre for Cancer Genetic Epidemiology, Department of Oncology, Strangeways Laboratory, University of Cambridge, Cambridge, CB1 8RN UK; 16 Department of Applied Health Research, University College London, London, WC1E 7HB UK; 17 Department of Medical Biochemistry and Genetics, Institute of Biomedicine, University of Turku, FI-20014 Turku, Finland; 18 Tyks Microbiology and Genetics, Department of Medical Genetics, Turku University Hospital, 20521 Turku, Finland; 19 BioMediTech, University of Tampere, 33520 Tampere, Finland; 20 Division of Nutritional Epidemiology, Institute of Environmental Medicine, Karolinska Institutet, SE-171 77 Stockholm, Sweden; 21 Division of Cancer Sciences, Manchester Academic Health Science Centre, Radiotherapy Related Research, Manchester NIHR Biomedical Research Centre, The Christie Hospital NHS Foundation Trust, University of Manchester, Manchester, M13 9PL UK; 22 Department of Epidemiology, Harvard School of Public Health, Boston, MA 02115 USA; 23 Program in Genetic Epidemiology and Statistical Genetics, Department of Epidemiology, Harvard T.HChan School of Public Health, Boston, MA 02115 USA; 24 GRC N°5 ONCOTYPE-URO, UPMC Univ Paris 06, Tenon Hospital, F-75020 Paris, France; 25 CeRePP, Tenon Hospital, F-75020 Paris, France; 26 Department of Molecular Medicine, Aarhus University Hospital, 8200 Aarhus N, Denmark; 27 Department of Clinical Medicine, Aarhus University, 8200 Aarhus N, Denmark; 28 Department of Medical Genetics, Oslo University Hospital, 0424 Oslo, Norway; 29 Department of Epidemiology, The University of Texas MD Anderson Cancer Center, Houston, TX 77030 USA; 30 Department of Oncology, Addenbrooke’s Hospital, University of Cambridge, Cambridge, CB2 0QQ UK; 31 Cancer Research UK Cambridge Research Institute, Li Ka Shing Centre, Cambridge, CB2 0RE UK; 32 Nuffield Department of Surgical Sciences, University of Oxford, Oxford, OX1 2JD UK; 33 School of Social and Community Medicine, University of Bristol, Canynge Hall, 39 Whatley Road, Bristol, BS8 2PS UK; 34 Cancer Epidemiology, Nuffield Department of Population Health, University of Oxford, Oxford, OX3 7LF UK; 35 Department of Surgical Oncology, Princess Margaret Cancer Centre, Toronto, ON M5G 2M9 Canada; 36 Department of Radiation Oncology, Icahn School of Medicine at Mount Sinai, New York, NY 10029 USA; 37 Department of Genetics and Genomic Sciences, Icahn School of Medicine at Mount Sinai, New York, NY 10029-5674 USA; 38 Centre for Molecular Oncology, Barts Cancer Institute, John Vane Science Centre, Queen Mary University of London, London, EC1M 6BQ UK; 39 Cancer Epidemiology and Intelligence Division, Cancer Council Victoria, Melbourne, VIC 3004 Australia; 40 Centre for Epidemiology and Biostatistics, Melbourne School of Population and Global Health, The University of Melbourne, Melbourne, VIC 3010 Australia; 41 Division of Urologic Surgery, Brigham and Womens Hospital, Boston, MA 02115 USA; 42 Fundación Pública Galega de Medicina Xenómica-SERGAS, Grupo de Medicina Xenómica, CIBERER, IDIS, 15706 Santiago de Compostela, Spain; 43 Department of Epidemiology, Gillings School of Global Public Health, University of North Carolina, Columbia, SC 29208 USA; 44 Centre for Research in Environmental Epidemiology (CREAL), Barcelona Institute for Global Health (ISGlobal), 08003 Barcelona, Spain; 45 CIBER Epidemiología y Salud Pública (CIBERESP), 28029 Madrid, Spain; 46 IMIM (Hospital del Mar Research Institute), 08003 Barcelona, Spain; 47 Universitat Pompeu Fabra (UPF), 08002 Barcelona, Spain; 48 Channing Division of Network Medicine, Department of Medicine, Brigham and Women’s Hospital/Harvard Medical School, Boston, MA 02184 USA; 49 Department of Cancer Epidemiology, Moffitt Cancer Center, Tampa, FL 33612 USA; 50 Division of Public Health Sciences, Fred Hutchinson Cancer Research Center, Seattle, WA 98109-1024 USA; 51 Department of Epidemiology, School of Public Health, University of Washington, Seattle, WA 98195 USA; 52 International Hereditary Cancer Center, Department of Genetics and Pathology, Pomeranian Medical University, 70-115 Szczecin, Poland; 53 Faculty of Health and Medical Sciences, University of Copenhagen, 2200 Copenhagen, Denmark; 54 Department of Clinical Biochemistry, Herlev and Gentofte Hospital, Copenhagen University Hospital, Herlev, 2200 Copenhagen, Denmark; 55 Division of Clinical Epidemiology and Aging Research, German Cancer Research Center (DKFZ), D-69120 Heidelberg, Germany; 56 German Cancer Consortium (DKTK), German Cancer Research Center (DKFZ), D-69120 Heidelberg, Germany; 57 Division of Preventive Oncology, German Cancer Research Center (DKFZ) and National Center for Tumor Diseases (NCT), 69120 Heidelberg, Germany; 58 Institute for Human Genetics, University Hospital Ulm, 89075 Ulm, Germany; 59 Department of Genitourinary Medical Oncology, The University of Texas MD Anderson Cancer Center, Houston, TX 77030 USA; 60 Department of Genetics, Portuguese Oncology Institute of Porto, 4200-072 Porto, Portugal; 61 Biomedical Sciences Institute (ICBAS), University of Porto, 4050-313 Porto, Portugal; 62 Department of Population Sciences, Beckman Research Institute of the City of Hope, Duarte, CA 91010 USA; 63 Faculty of Medicine and Health Sciences, Basic Medical Sciences, Ghent University, B-9000 Gent, Belgium; 64 Faculty of Medicine, Department of Surgery, University of Malaya, 50603 Kuala Lumpur, Malaysia; 65 Department of Urology, University of Washington, Seattle, WA 98195 USA; 66 Institute of Human Genetics, University Medical Center Hamburg-Eppendorf, D-20246 Hamburg, Germany; 67 Molecular Medicine Center, Department of Medical Chemistry and Biochemistry, Medical University of Sofia, 1431 Sofia, Bulgaria; 68 Department of Oncology, Cross Cancer Institute, University of Alberta, Edmonton, AB T6G 1Z2 Canada; 69 Division of Radiation Oncology, Cross Cancer Institute, Edmonton, AB T6G 1Z2 Canada; 70 Molecular Endocrinology Laboratory, Department of Cellular and Molecular Medicine, KU Leuven, BE-3000 Leuven, Belgium; 71 Manchester Cancer Research Centre, Faculty of Biology Medicine and Health, Manchester Academic Health Science Centre, NIHR Manchester Biomedical Research Centre, Health Innovation Manchester, University of Manchester, Manchester, M13 9WL UK; 72 Genomic Medicine Group, Galician Foundation of Genomic Medicine, Instituto de Investigacion Sanitaria de Santiago de Compostela (IDIS), Complejo Hospitalario Universitario de Santiago, Servicio Galego de Saúde, SERGAS, 15706 Santiago de Compostela, Spain; 73 Moores Cancer Center, University of California San Diego, La Jolla, CA 92037 USA; 74 Department of Urology, Erasmus University Medical Center, 3015 CE Rotterdam, The Netherlands; 75 Cancer and Environment Group, Center for Research in Epidemiology and Population Health (CESP), INSERM, University Paris-Sud, University Paris-Saclay, 94807 Villejuif Cédex, France; 76 Clinical Gerontology Unit, University of Cambridge, Cambridge, CB2 2QQ UK; 77 Division of Genetic Epidemiology, Department of Medicine, University of Utah School of Medicine, Salt Lake City, UT 84112 USA; 78 George EWahlen Department of Veterans Affairs Medical Center, Salt Lake City, UT 84148 USA; 79 The University of Surrey, Guildford, Surrey, GU2 7XH UK; 80 Department of Laboratory Medicine and Pathology, Mayo Clinic, Rochester, MN 55905 USA; 81 Division of Biomedical Statistics and Informatics, Mayo Clinic, Rochester, MN 55905 USA; 82 Royal Marsden NHS Foundation Trust, London, SW3 6JJ UK; 83 Molecular Cancer Epidemiology Laboratory, QIMR Berghofer Institute of Medical Research, Herston, QLD 4006 Australia; 84 School of Medicine, University of Queensland, Herston, QLD 4006 Australia; 85 Royal Brisbane and Women’s Hospital, Herston, QLD 4029 Australia; 86 The Kinghorn Cancer Centre (TKCC), Victoria, NSW 2010 Australia; 87 Prostate Cancer Research Group, South Australian Health and Medical Research Institute, Adelaide, SA 5000 Australia; 88 Department of Physiology, Biomedicine Discovery Institute, Cancer Program, Monash University, Melbourne, VIC 3800 Australia; 89 University of Adelaide, North Terrace, Adelaide, SA 5005 Australia; 90 Fimlab Laboratories, Tampere University Hospital, FI-33520 Tampere, Finland; 91 Finnish Cancer Registry, FI-00130 Helsinki, Finland; 92 Department of Urology, Helsinki University Central Hospital and University of Helsinki, FI-00014 Helsinki, Finland; 93 Division of Biostatistics and Bioinformatics, University of Maryland Greenebaum Cancer Center, and Department of Epidemiology and Public Health, University of Maryland School of Medicine, Baltimore, MD 21201 USA; 94 Cancer Genomics Research Laboratory (CGR), Division of Cancer Epidemiology and Genetics, FNLCR Leidos Biomedical Research, National Cancer Institute, Frederick, MD 21701 USA; 95 Division of Cancer Epidemiology and Genetics, DNA Extraction and Staging Laboratory (DESL), Cancer Genomics Research Laboratory (CGR), FNLCR Leidos Biomedical Research, National Cancer Institute, Frederick, MD 21701 USA; 96 Sheffield Institute for Nucleic Acids, University of Sheffield, Sheffield, S10 2TN UK; 97 Cambridge Cancer Trials Centre, Cambridge Clinical Trials Unit-Cancer Theme, Cambridge University Hospitals NHS Foundation Trust, Cambridge, CB2 0QQ UK; 98 Department of Medical Imaging, University Health Network, Toronto, ON M5G 2C4 Canada; 99 Department of Pathology, University Health Network, Toronto, ON M5G 2C4 Canada; 100 Advanced Radiation Biology Research Program, Research Center for Charged Particle Therapy, National Institute of Radiological Sciences, Chiba, 263-8555 Japan; 101 Department of Urology, National Hospital Organization Tokyo Medical Center, Tokyo, 152-8902 Japan; 102 Department of Urology, Nanfang Hospital, Southern Medical University, 510515 Guangzhou, China; 103 Department of Pathology, The First Affiliated Hospital, Zhejiang University Medical College, 310009 Hangzhou, China; 104 Department of Pathology, Changhai Hospital, The Second Military Medical University, 200433 Shanghai, China; 105 Department of Urology, First Affiliated Hospital, Medical College, Zhengzhou University, 450003 Zhengzhou, China; 106 Department of Urology, North Sichuan Medical College, 637000 Nanchong, China; 107 Department of Nutrition Science, Shenyang Medical College, 110034 Shenyang, China; 108 Tissupath Pty Ltd., Melbourne, VIC 3122 Australia; 109 Department of Medical Physics, Complexo Hospitalario Universitario de Santiago, SERGAS, 15706 Santiago de Compostela, Spain; 110 Urology Department, Hospital Germans Trias I Pujol, 08916 Barcelona, Spain; 111 Laboratory and Department of Urology, Hospital Clínic, Institut d’Investigacions Biomèdiques August Pi i Sunyer (IDIBAPS), Universitat de Barcelona, 08036 Barcelona, Spain; 112 Centre de Recerca Biomèdica CELLEX, 08036 Barcelona, Spain; 113 Department and Laboratory of Urology, Hospital Clínic, Institut d’Investigacions Biomèdiques August Pi i Sunyer (IDIBAPS), Universitat de Barcelona, 08036 Barcelona, Spain; 114 Genitourinary Program, Moffitt Cancer Center, Tampa, FL 33612 USA; 115 Department of Urology, Klinikum rechts der Isar der Technischen Universitaet Muenchen, 81675 Munich, Germany; 116 Department of General and Clinical Pathology, Alexandrovska University Hospital, Medical University, 1431 Sofia, Bulgaria; 117 Center of Excellence in Genomic Medicine Research, King Abdulaziz University, Jeddah, 2252 3270 Saudi Arabia; 118 Grupo de Medicina Xenómica, CIBERER, CIMUS, Universidad de Santiago de Compostela, Avenida de Barcelona, 15782 Santiago de Compostela, Spain; 119 Hérault Cancer Registry, Montpellier cedex 5, Montpellier, 34298 France; 120 Urology Department, Clinique Beau Soleil, 34070 Montpellier, France; 121 INSERM U1147, 75013 Paris, France; 122 Department of Clinical Science, Intervention and Technology, Karolinska Institutet, SE-171 77 Stockholm, Sweden; 123 Swedish Agency for Health Technology Assessment and Assessment of Social Services, SE-102 33 Stockholm, Sweden; 124 Department of Surgical and Perioperative Sciences, Urology and Andrology, Umeå University, SE-901 85 Umeå, Sweden; 125 Department of Surgical Sciences, Uppsala University, SE-751 85 Uppsala, Sweden; 126 Faculty of Medicine and Health, Department of Urology, Örebro University, SE-701 82 Örebro, Sweden; 127 Medical Research Council (MRC) Integrative Epidemiology Unit, University of Bristol, Bristol, BS8 2BN UK; 128 National Institute for Health Research (NIHR) Biomedical Research Centre, University of Bristol, Bristol, BS8 1TH UK; 129 Department of Urology, Cancer Therapy and Research Center, University of Texas Health Science Center at San Antonio, San Antonio, TX 78229 USA; 130 Menzies Health Institute Queensland, Griffith University, Gold Coast, QLD 4222 Australia; 131 Cancer Council Queensland, Fortitude Valley, QLD 4006 Australia; 132 Chris O’Brien Lifehouse (COBLH), Camperdown, Sydney, NSW 2010 Australia; 133 Garvan Institute of Medical Research, Sydney, NSW 2010 Australia; 134 Dame Roma Mitchell Cancer Research Centre, University of Adelaide, Adelaide, SA 5005 Australia; 135 Department of Anatomy and Developmental Biology, Biomedicine Discovery Institute, Monash University, Melbourne, VIC 3800 Australia; 136 Prostate Cancer Translational Research Program, Cancer Research Division, Peter MacCallum Cancer Centre, Melbourne, VIC 3000 Australia; 137 Department of Molecular Medicine and Surgery, Karolinska Institutet, and Department of Urology, Karolinska University Hospital, 171 76 Stockholm, Sweden; 138 Department of Clinical Sciences at Danderyd Hospital, Karolinska Institutet, 182 88 Stockholm, Sweden; 139 Cancer Genome Project, Wellcome Trust Sanger Institute, Hinxton, Cambridge, CB10 1SA UK; 140 Department of Urology, Tampere University Hospital, University of Tampere, Kalevantie 4, FI-33014 Tampere, Finland; 141 Faculty of Medicine and Life Sciences, University of Tampere, FI-33014 Tampere, Finland; 142 Department of Epidemiology, School of Health Sciences, University of Tampere, FI-33014 Tampere, Finland; 143 University of Cambridge Department of Oncology, Oncology Centre, Cambridge University Hospitals NHS Foundation Trust, Cambridge, CB1 8RN UK; 144 Washington University School of Medicine, StLouis, MO 63110 USA; 145 Department of Urology, Aarhus University Hospital, 8200 Aarhus N, Denmark; 146 Department of Radiation Oncology, University of Rochester Medical Center, Rochester, NY 14620 USA; 147 Professor of Pathology and Pediatrics, Albert Einstein College of Medicine, Bronx, NY 10461 USA; 148 Second Military Medical University, 200433 Shanghai, China; 149 Wuxi Second Hospital, Nanjing Medical University, 214003 Wuxi, Jiangzhu China; 150 Department of Urology, The First Affiliated Hospital, Chongqing Medical University, 200032 Chongqing, China; 151 The People’s Hospital of Liaoning Province and The People’s Hospital of China Medical University, 110001 Shenyang, China; 152 Department of Urology, Zhongshan Hospital, Fudan University Medical College, 200032 Shanghai, China; 153 Precision Medicine, School and Clinical Sciences at Monash Health, Monash University, Clayton, VIC 3168 Australia; 154 Menzies Institute for Medical Research, University of Tasmania, Hobart, TAS 7000 Australia; 155 Faculty of Medical Science, John Radcliffe Hospital, University of Oxford, Oxford, OX1 2JD UK; 156 Department of Radiation Oncology, Complexo Hospitalario Universitario de Santiago, SERGAS, 15706 Santiago de Compostela, Spain; 157 Division of Family Medicine, Department of Neurobiology, Care Science and Society, Karolinska Institutet, Huddinge, SE-171 77 Stockholm, Sweden; 158 Scandinavian Development Services, 182 33 Danderyd, Sweden; 159 University of Cantabria-IDIVAL, 39005 Santander, Spain; 160 School of Public Health, Louisiana State University Health Sciences Center, New Orleans, LA 70112 USA; 161 National Human Genome Research Institute, National Institutes of Health, Bethesda, MD 20892 USA; 162 Department of Urology, Herlev and Gentofte Hospital, Copenhagen University Hospital, Herlev, 2200 Copenhagen, Denmark; 163 Copenhagen Prostate Cancer Center, Department of Urology, Rigshospitalet, Copenhagen University Hospital, DK-2730 Herlev, Denmark; 164 Saarland Cancer Registry, 66119 Saarbrücken, Germany; 165 Department of Urology, University Hospital Ulm, 89075 Ulm, Germany; 166 Cancer Prevention Institute of California, Fremont, CA 94538 USA; 167 Department of Health Research and Policy (Epidemiology) and Stanford Cancer Institute, Stanford University School of Medicine, Stanford, CA 94305-5101 USA; 168 Department of Radiotherapy, Ghent University Hospital, B-9000 Gent, Belgium; 169 Cancer Research Malaysia (CRM), Outpatient Centre, Subang Jaya Medical Centre, 47500 Subang Jaya, Selangor Malaysia; 170 Division of Medical Oncology, Urogenital Unit, Department of Oncology at the University Hospital Centre Zagreb, Šalata 2, 10000 Zagreb, Croatia; 171 Department of Urology, University Hospital Center Zagreb, University of Zagreb School of Medicine, Šalata 2, 10000 Zagreb, Croatia; 172 Department of Urology and Alexandrovska University Hospital, Medical University of Sofia, 1431 Sofia, Bulgaria; 173 Department of Urology, University Hospitals Leuven, BE-, 3000 Leuven, Belgium; 174 Southampton General Hospital, The University of Southampton, Southampton, SO16 6YD UK; 175 Genetic Oncology Unit, CHUVI Hospital, Complexo Hospitalario Universitario de Vigo, Instituto de Investigación Biomédica Galicia Sur (IISGS), 36204 Vigo (Pontevedra), Spain; 176 Moores Cancer Center, Department of Family Medicine and Public Health, University of California San Diego, La Jolla, CA 92093-0012 USA; 177 Department of Clinical Chemistry, Erasmus University Medical Center, 3015 CE Rotterdam, The Netherlands; 178 Program for Personalized Cancer Care, NorthShore University Health System, Evanston, IL 60201 USA; 179 Department of Epidemiology, University of Washington, Seattle, WA 98195 USA; 180 Department of Epidemiology and Biostatistics, School of Public Health, Imperial College, London, SW7 2AZ UK; 181 Genomics England, Queen Mary University of London, Dawson Hall, Charterhouse Square, London, EC1M 6BQ UK; 182 Genomic Epidemiology Group, German Cancer Research Center (DKFZ), D-69120 Heidelberg, Germany; 183 Epidemiology Program, University of Hawaii Cancer Center, Honolulu, HI 96813 USA; 184 Dana-Farber Cancer Institute, Boston, MA 02215 USA; 185 Paris-Sud University, UMRS 1018, Cedex 94807 Villejuif, France

Correction to: *Nature Communications*; 10.1038/s41467-018-06863-1, published online 5 November 2018.

The original version of this Article contained an error in the spelling of the author Manuela Gago-Dominguez, which was incorrectly given as Manuela G. Dominguez. This has now been corrected in both the PDF and HTML versions of the Article.

