# Supplementary material for: Germline variation at 8q24 and prostate cancer risk in men of European ancestry
Source: Nat Commun. 2018 Nov 5;9:4616. doi: 10.1038/s41467-018-06863-1 (PMC6218483; doi:10.1038/s41467-018-06863-1)
Supplement: Supplementary file 1 — Supplementary Information [file 41467_2018_6863_MOESM1_ESM.pdf]

Germline Variation at 8q24 and Prostate Cancer Risk in Men of European Ancestry

Matejcic et al.

Supplementary Figure 1 - Full Region

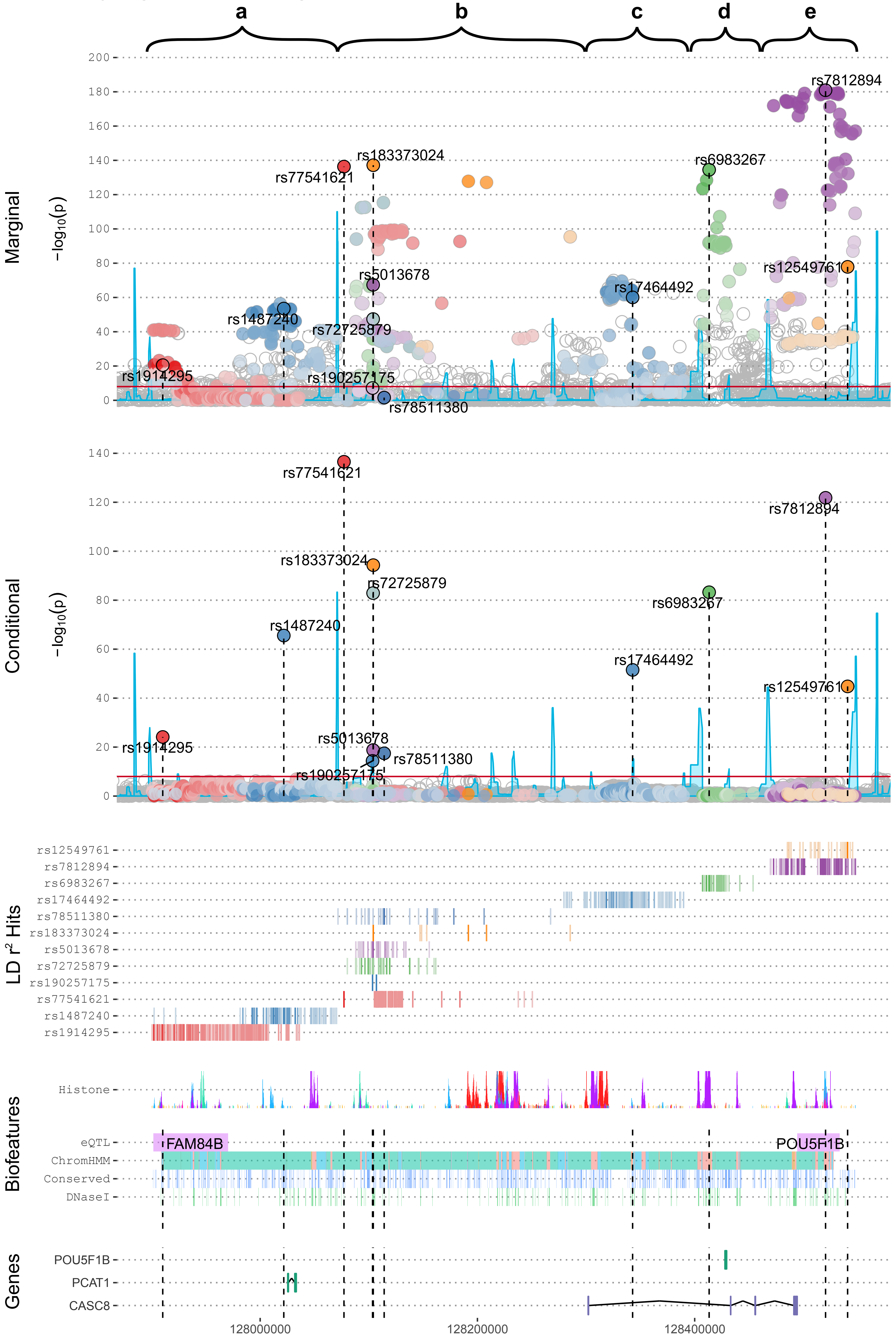

Supplementary Figure 1 - Subregion a

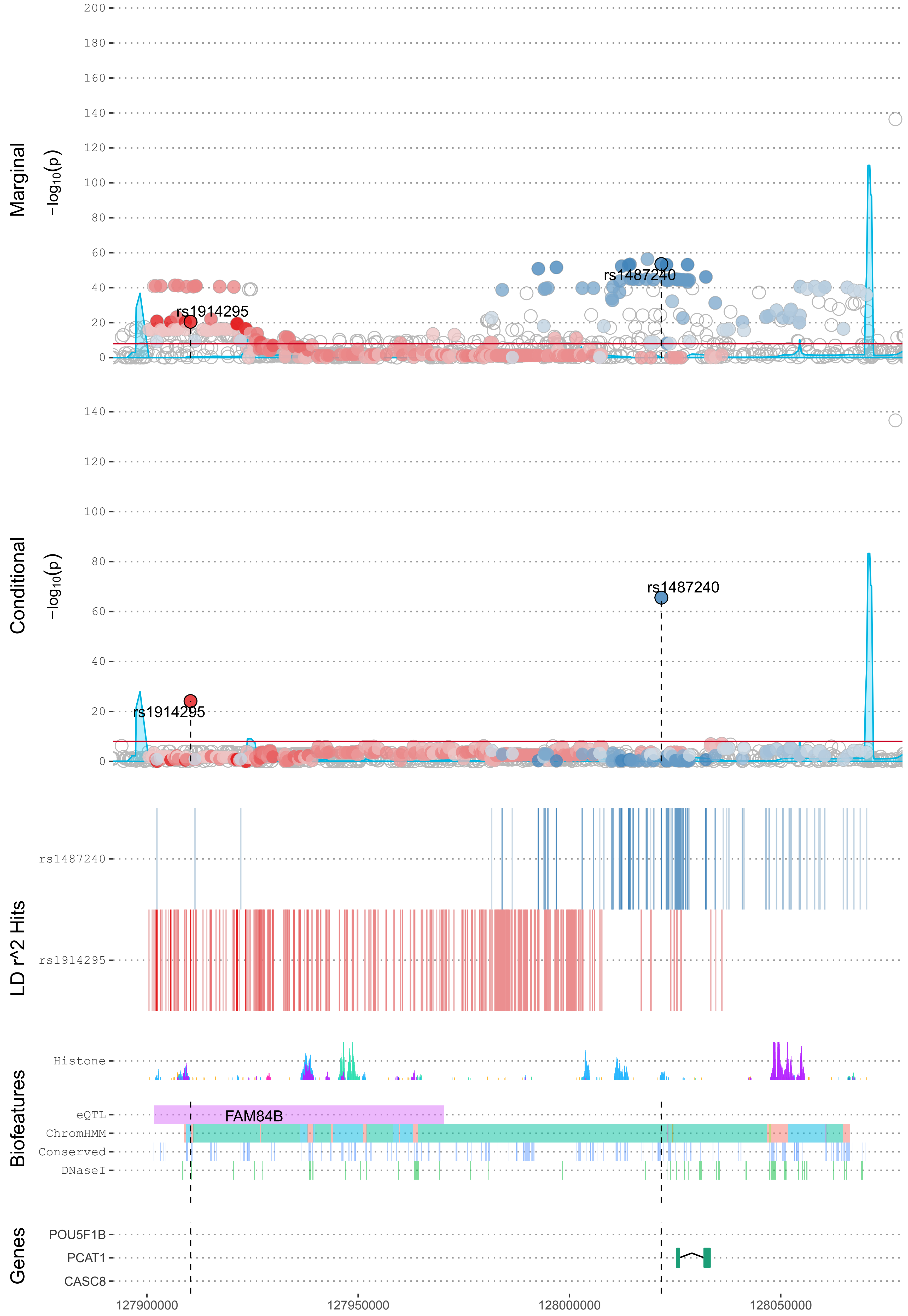

Supplementary Figure 1 - Subregion b

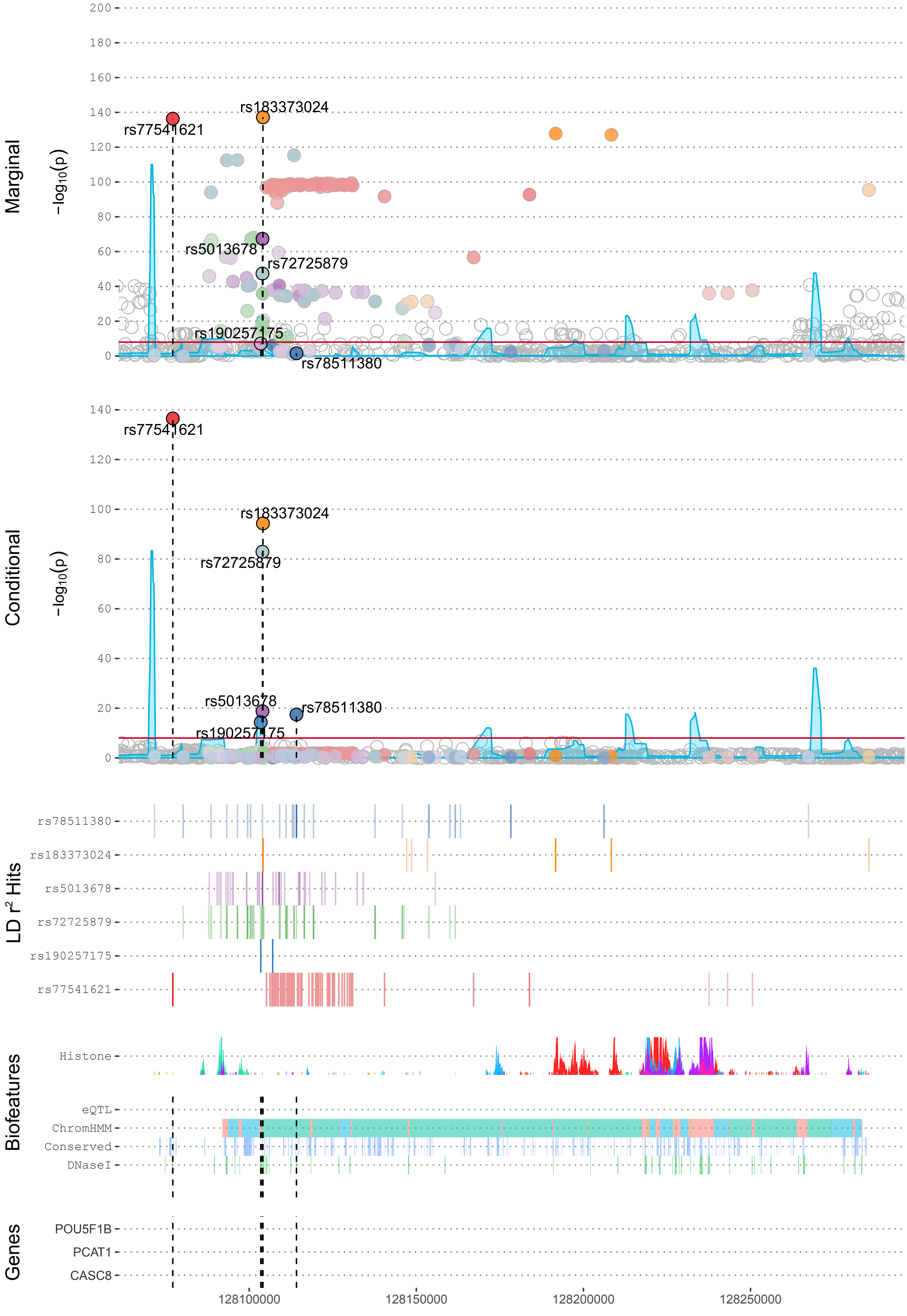

Supplementary Figure 1 - Subregion c

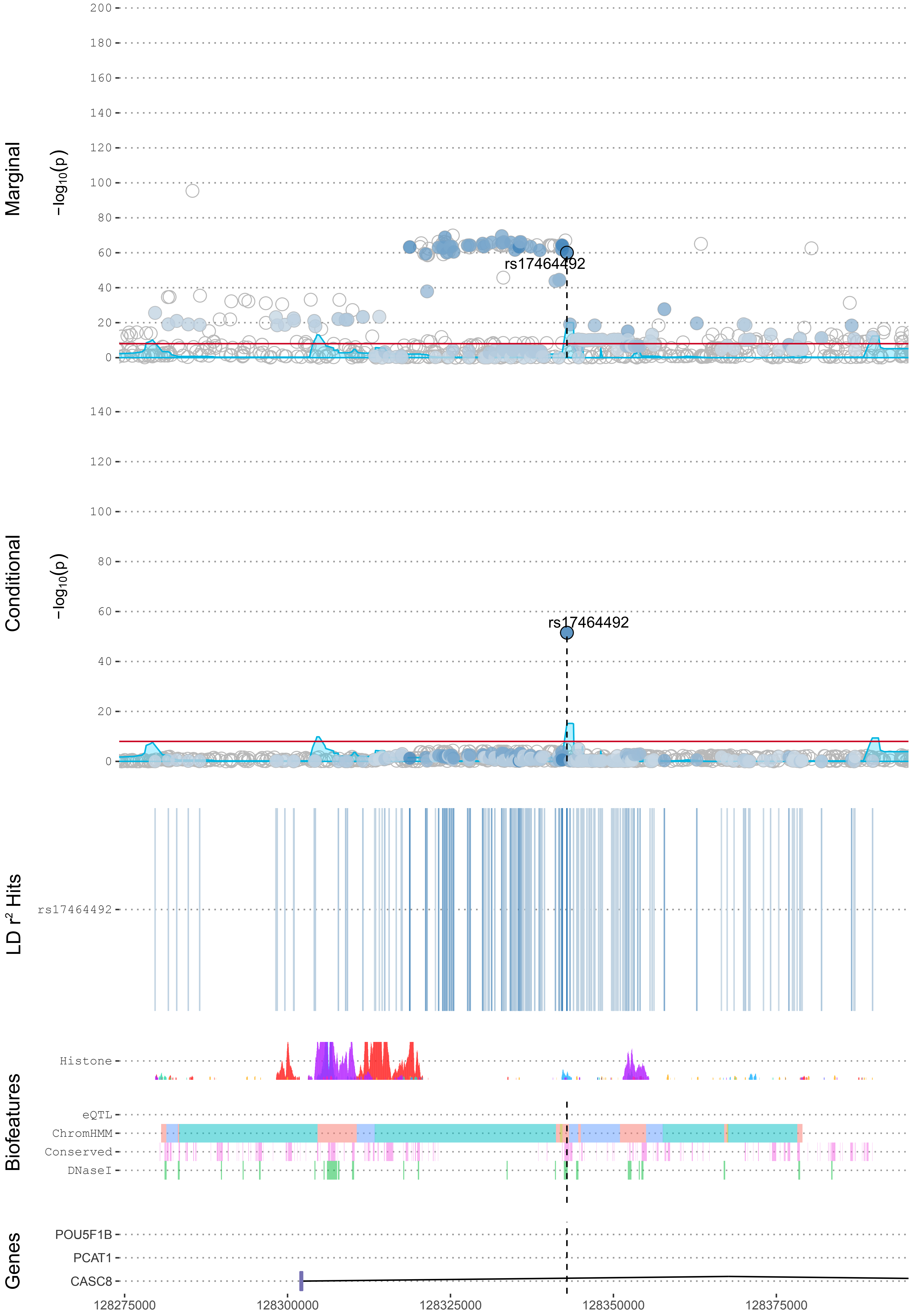

Supplementary Figure 1 - Subregion d

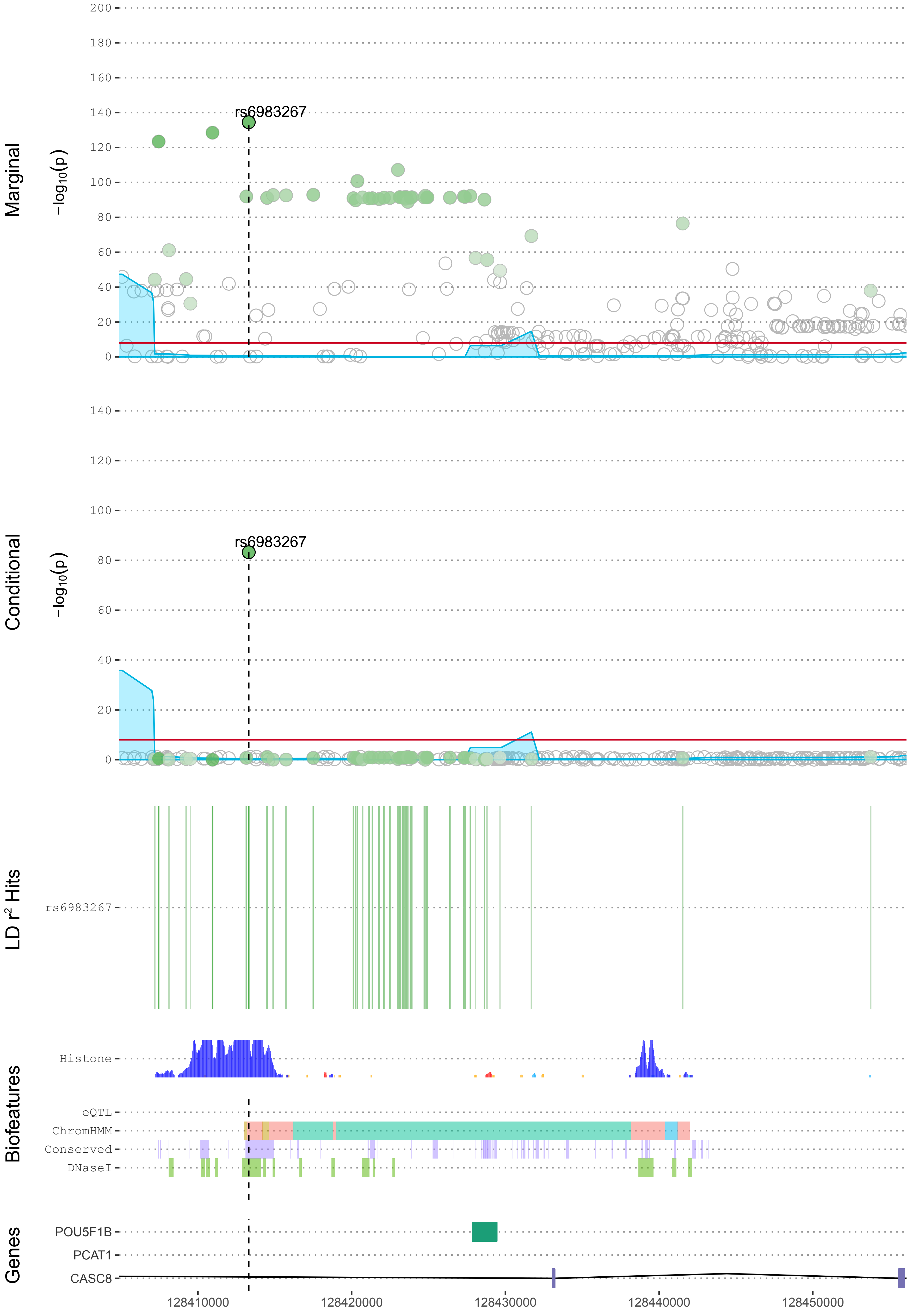

Supplementary Figure 1 - Subregion e

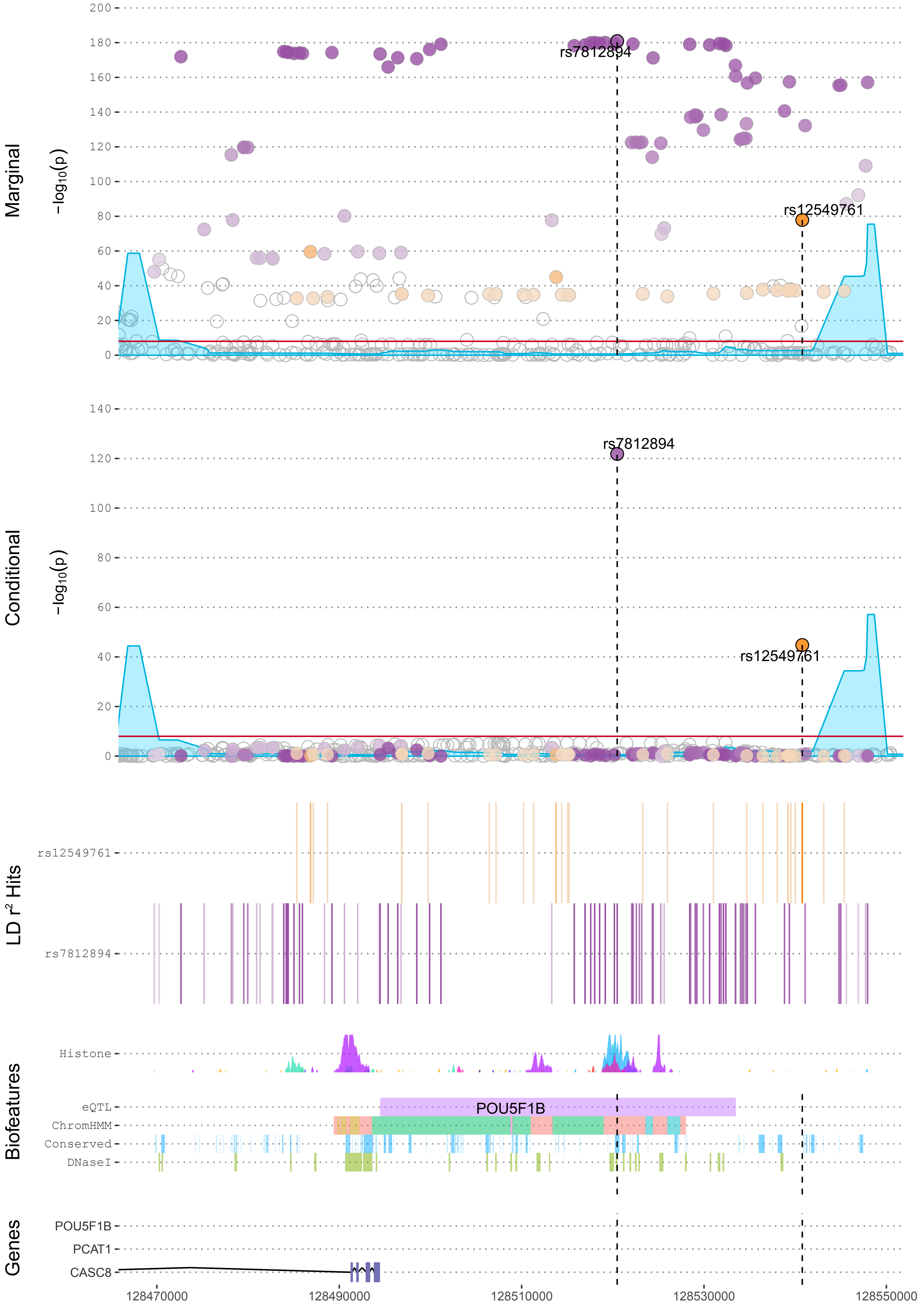

Supplementary Figure 1. LocusExplorer plots of the 12 independent signals at 8q24 by subregion. Plots are shown for all 12 hits combined and divided into five subregions (a-e) to provide greater resolution of the context of individual association signals. 'Marginal' and 'Conditional' Manhattan plot panels show marginal and conditional association results respectively. Variant positions (x-axis) and $-\log_{10}$ *P*-values from Wald test (y-axis) are shown, with the red line indicating the threshold for genome-wide significant association with PCa risk ($P \leq 5x10^{-8}$) and blue peaks local estimates of recombination rates. The position of the 12 independent variants within the positional boundaries is labeled in each plot. Clusters of correlated variants for each independent signal are distinguished using different colors and also depicted on the 'LD r^2 Hits' track. Stronger shading indicates greater correlation with the lead variant, with variants not correlated at $r^2 \geq 0.2$ with any lead variant uncolored. Pairwise correlations are based on the European ancestry (EUR) panel from the 1000 Genomes Project (1KGP) Phase 3. The relative position of RefSeq genes and biological annotations are shown in the 'Genes' and 'Biofeatures' panels respectively. Genes on the positive strand are denoted in green and those on the negative strand in purple. Annotations displayed are: histone modifications in ENCODE tier 1 cell lines (Histone track), the positions of any variants that were eQTLs with prostate tumor expression in TCGA prostate adenocarcinoma samples and the respective genes for which expression is altered (eQTL track), chromatin state categorizations in the PrEC cell-line by ChromHMM (ChromHMM track), the position of conserved element peaks (Conserved track) and the position of DNaseI hypersensitivity site peaks in ENCODE prostate cell-lines (DNaseI track). The data displayed in this plot may be explored interactively through the LocusExplorer application (http://www.oncogenetics.icr.ac.uk/8q24/).

Supplementary Figure 2

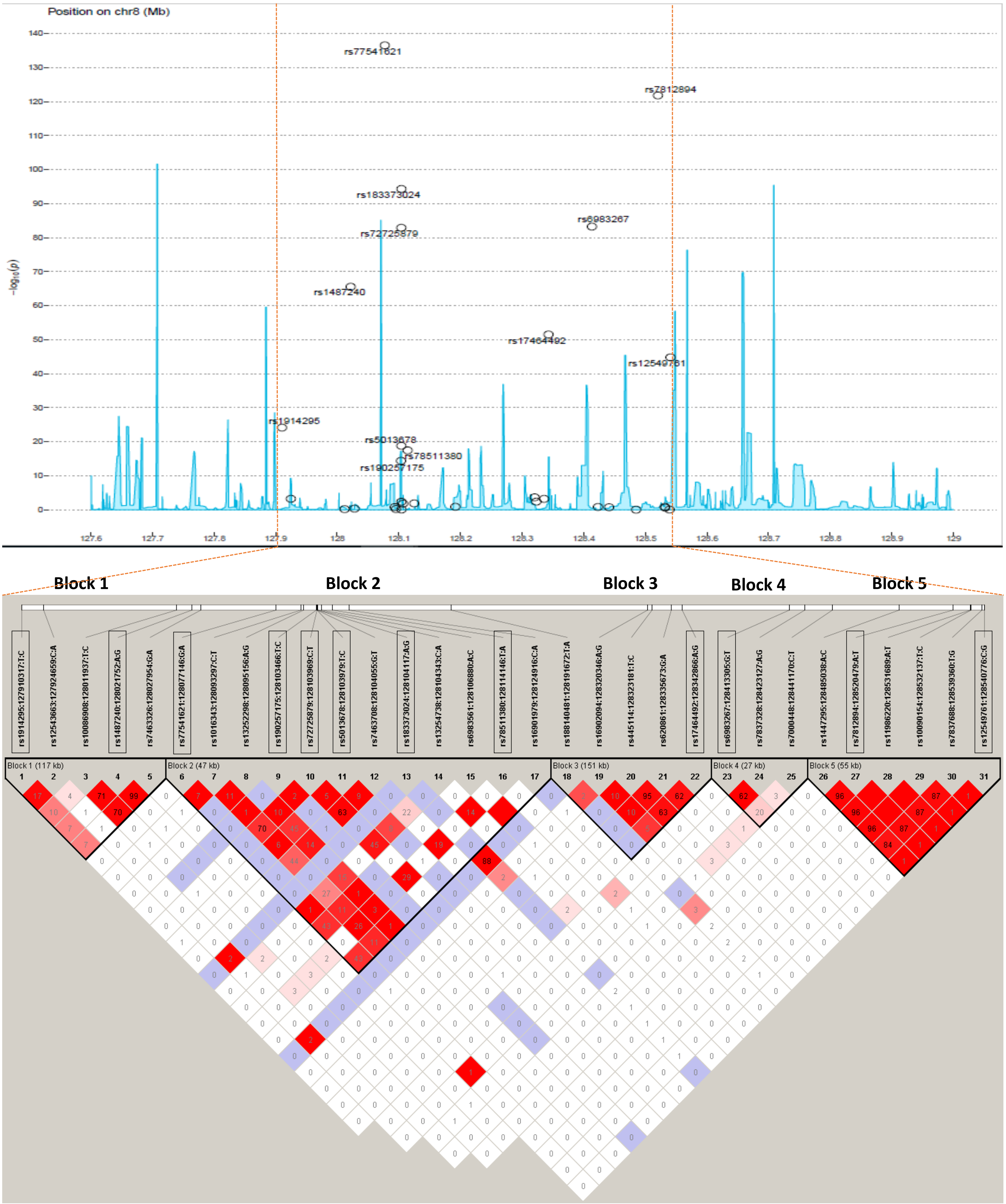

Supplementary Figure 2. Linkage disequilibrium plot of the 12 independent signals and previously published PCa risk variants within the 8q24 region. The upper panel shows variants plotted by position (x-axis) and -log10 *P*-value from Wald test (y-axis). The blue peaks indicate local estimates of recombination rates. The 12 independent risk variants are labeled in the plot. The lower panel shows the LD plot of the 8q24 region bounded by rs1914295 and rs12549761 (chr8:127910317-128540776). The 12 independent variants are surrounded by squares. D clusters and correlation coefficients (r^2 and D') were inferred based on recombination hotspots using Haploview 4.2 [29]. Pairwise correlations were based on the European ancestry (EUR) panel from the 1000 Genomes Project (1KGP) Phase 3. Colors represent D' values while numbers represent r^2 values. Triangles define major LD blocks across the 8q24 region.

Supplementary Table 1. Association of common haplotypes at 8q24 with PCa risk under the additive model[1]

Haplotype blocks[1]	Frequency	OR (95% CI)[2]	p-value
rs1914295 - rs1487240			
C - G	0.019	1.00 (Ref)	
C - **A**	0.291	1.16 (1.05-1.27)	$2.19x10^{-3}$
**T** - G	0.218	1.10 (0.99-1.21)	$5.89x10^{-2}$
**T** - **A**	0.472	1.32 (1.21-1.45)	$1.10x10^{-9}$
rs77541621 - rs190257175 - rs72725879 - rs5013678 - rs183373024 - rs78511380			
G - **T** - C - C - A - **T**	0.197	1.00 (Ref)	
G - **T** - C - **T** - A - **T**	0.556	1.11 (1.08-1.14)	$7.85x10^{-17}$
G - **T** - C - **T** - **G** - **T**	0.013	3.41 (3.06-3.80)	$1.52x10^{-110}$
G - **T** - **T** - **T** - A - A	0.079	1.21 (1.16-1.26)	$1.18x10^{-19}$
G - **T** - **T** - **T** - A - **T**	0.111	1.44 (1.39-1.49)	$4.96x10^{-86}$
**A** - **T** - C - **T** - A - **T**	0.035	2.10 (1.98-2.23)	$4.46x10^{-127}$
G - C - **T** - **T** - A - **T**	0.005	0.84 (0.73-0.95)	$7.34x10^{-3}$
rs7812894 - rs12549761			
T - G	0.108	1.00 (Ref)	
T - **C**	0.768	1.22 (1.18-1.26)	$1.30x10^{-37}$
**A** - C	0.122	1.76 (1.69-1.83)	$3.10x10^{-162}$

[1]Haplotypes were constructed using the 12 variants from the final stepwise model.

[2]Letters separated by hyphens correspond to the nucleotide of each variant in the corresponding sequence with risk alleles highlighted in bold.

[3]Estimated effect of each haplotype relative to the reference haplotype (the one with the highest number of non-risk alleles) adjusted for country and 7 principal components from the OncoArray dataset.

Supplementary Table 2. Marginal estimates for previously reported PCa risk variants at 8q24 in the current study[1]

Variant ID	Position[2]	Allele[3]	RAF[4]	LD cluster[5]	OR (95%CI)[6]	p-value	Source study[7]
rs12543663	127924659	C/A	0.29	block 1	1.13 (1.11-1.15)	$8.38x10^{-40}$	Al Olama *et al*, 2009 (Nat Genet)
rs10086908	128011937	T/C	0.68	block 1	1.14 (1.12-1.16)	$1.69x10^{-44}$	Al Olama *et al*, 2009 (Nat Genet)
rs7463326	128027954	G/A	0.75	block 1	1.16 (1.14-1.18)	$6.70x10^{-54}$	Conti *et al*, 2017 (JNCI)
rs77541621	128077146	A/G	0.02	block 2	1.83 (1.74-1.92)	$4.33x10^{-137}$	Hoffmann *et al*, 2015 (Cancer Discov)
rs1016343	128093297	T/C	0.22	block 2	1.25 (1.23-1.28)	$3.76x10^{-113}$	Al Olama *et al*, 2009 (Nat Genet)
rs13252298	128095156	A/G	0.71	block 2	1.14 (1.12-1.16)	$1.65x10^{-43}$	Al Olama *et al*, 2009 (Nat Genet)
rs72725879	128103969	T/C	0.2	block 2	1.17 (1.14-1.19)	$3.96x10^{-48}$	Han *et al*, 2016 (JNCI)
rs7463708	128104055	T/G	0.28	block 2	1.13 (1.1-1.15)	$1.58x10^{-36}$	Marzec *et al*, 2016 (Oncotarget)
rs183373024	128104117	G/A	0.01	block 2	3.2 (2.92-3.5)	$6.60x10^{-138}$	Gudmundsson *et al*, 2012 (Nat Genet)
rs13254738	128104343	C/A	0.33	block 2	1.08 (1.06-1.09)	$3.68x10^{-16}$	Haiman *et al*, 2007 (Nat Genet)
rs6983561	128106880	C/A	0.04	block 2	1.59 (1.52-1.66)	$3.44x10^{-99}$	Haiman *et al*, 2007 (Nat Genet)
rs16901979	128124916	A/C	0.04	block 2	1.59 (1.53-1.66)	$1.60x10^{-99}$	Haiman *et al*, 2007 (Nat Genet)
rs188140481	128191672	A/T	0.01	block 3	3.18 (2.89-3.49)	$1.61x10^{-128}$	Gudmundsson *et al*, 2012 (Nat Genet)
rs16902094	128320346	G/A	0.16	block 3	1.22 (1.19-1.24)	$4.95x10^{-64}$	Gudmundsson *et al*, 2009 (Nat Genet)
rs445114	128323181	T/C	0.64	block 3	1.16 (1.14-1.18)	$8.29x10^{-64}$	Gudmundsson *et al*, 2009 (Nat Genet)
rs620861	128335673	G/A	0.64	block 3	1.16 (1.14-1.18)	$7.08x10^{-67}$	Al Olama *et al*, 2009 (Nat Genet)
rs17464492	128342866	A/G	0.72	block 3	1.17 (1.14-1.19)	$9.05x10^{-61}$	Al Olama *et al*, 2009 (Nat Genet)
rs6983267	128413305	G/T	0.5	block 4	1.23 (1.21-1.25)	$3.15x10^{-135}$	Haiman *et al*, 2007 (Nat Genet)
rs7837328	128423127	A/G	0.38	block 4	1.19 (1.17-1.21)	$2.80x10^{-92}$	Yeager *et al*, 2007 (Nat Genet)
rs7000448	128441170	T/C	0.4	block 4	1.08 (1.06-1.1)	$3.55x10^{-19}$	Haiman *et al*, 2007 (Nat Genet)
rs1447295	128485038	A/C	0.1	block 5	1.44 (1.4-1.48)	$1.55x10^{-174}$	Amundadottir *et al*, 2006 (Nat Genet)
rs7812894	128520479	A/T	0.1	block 5	1.45 (1.41-1.49)	$1.20x10^{-181}$	Conti *et al*, 2017 (JNCI)
rs11986220	128531689	A/T	0.1	block 5	1.45 (1.41-1.49)	$3.35x10^{-180}$	Jia *et al*, 2009 (PLoS Genet)
rs10090154	128532137	T/C	0.1	block 5	1.45 (1.41-1.49)	$5.90x10^{-180}$	Haiman *et al*, 2007 (Nat Genet)
rs7837688	128539360	T/G	0.11	block 5	1.42 (1.38-1.45)	$3.56x10^{-158}$	Yeager *et al*, 2007 (Nat Genet)
rs12549761	128540776	C/G	0.89	block 5	1.28 (1.25-1.31)	$1.38x10^{-78}$	Conti *et al*, 2017 (JNCI)

[1]Risk estimates were derived from a meta-analysis of marginal results of OncoArray and iCOGS data.
[2]Chromosome 8 co-ordinates based on human genome build 37 (GRCh37).
[3]Risk allele/reference allele.
[4]Risk allele frequency.
[5]LD clusters were inferred based on recombination hotspots using Haploview 4.2.
[6]Per-allele odds ratio and 95% confidence interval adjusted for country and 7(OncoArray)/8(iCOGS) principal components.
[7]Author, year (Journal) of original study reporting a genome-wide significant association between the genetic marker and PCa risk.

Supplementary Note 1. Funding & Acknowledgements. Information and acknowledgements for consortia contributing to the meta-analysis and for individual study groups within the PRACTICAL Consortium.

GWAS Studies in the Meta-Analysis Dataset

The authors wish to pay tribute to Brian Henderson, who was a driving force behind the OncoArray project, for his vision and leadership, and sadly passed away before seeing its fruition.

<u>OncoArray</u>
Genotyping of the OncoArray was funded by the US National Institutes of Health (NIH) [U19 CA 148537 for ELucidating Loci Involved in Prostate cancer SuscEptibility (ELLIPSE) project and X01HG007492 to the Center for Inherited Disease Research (CIDR) under contract number HHSN268201200008I] and by Cancer Research UK grant A8197/A16565. Additional analytic support was provided by NIH NCI U01 CA188392 (PI: Schumacher).

This study would not have been possible without the contributions of the following: Coordination team, bioinformatician and genotyping centers

Genotyping at CCGE, Cambridge: Craig Luccarini, Caroline Baynes, Patricia Harrington and Don Conroy

<u>iCOGS</u>
Funding for the iCOGS infrastructure came from: the European Community's Seventh Framework Programme under grant agreement n° 223175 (HEALTH-F2-2009-223175) (COGS), Cancer Research UK (C1287/A10118, C1287/A 10710, C12292/A11174, C1281/A12014, C5047/A8384, C5047/A15007, C5047/A10692, C8197/A16565), the National Institutes of Health (CA128978) and Post-Cancer GWAS initiative (1U19 CA148537, 1U19 CA148065 and 1U19 CA148112 - the GAME-ON initiative), the Department of Defence (W81XWH-10-1-0341), the Canadian Institutes of Health Research (CIHR) for the CIHR Team in Familial Risks of Breast Cancer, Komen Foundation for the Cure, the Breast Cancer Research Foundation, and the Ovarian Cancer Research Fund.

This study would not have been possible without the contributions of the following: Per Hall (COGS); Douglas F. Easton, Paul Pharoah, Kyriaki Michailidou, Manjeet K. Bolla, Qin Wang (BCAC), Andrew Berchuck (OCAC), Rosalind A. Eeles, Douglas F. Easton, Ali Amin Al Olama, Zsofia Kote-Jarai, Sara Benlloch (PRACTICAL), Georgia Chenevix-Trench, Antonis Antoniou, Lesley McGuffog, Fergus Couch and Ken Offit (CIMBA), Joe Dennis, Alison M. Dunning, Andrew Lee, and Ed Dicks, Craig Luccarini and the staff of the Centre for Genetic Epidemiology Laboratory, Javier Benitez, Anna Gonzalez-Neira and the staff of the CNIO genotyping unit, Jacques Simard and Daniel C. Tessier, Francois Bacot, Daniel Vincent, Sylvie LaBoissière and Frederic Robidoux and the staff of the McGill University and Génome Québec Innovation Centre, Stig E. Bojesen, Sune F. Nielsen, Borge G. Nordestgaard, and the staff of the Copenhagen DNA laboratory, and Julie M. Cunningham, Sharon A. Windebank, Christopher A. Hilker, Jeffrey Meyer and the staff of Mayo Clinic Genotyping Core Facility.

Additional Funding and Acknowledgments from Studies in the PRACTICAL Consortium

Information regarding the PRACTICAL consortium can be found at http://practical.icr.ac.uk

Aarhus

This study was supported by Innovation Fund Denmark, the Danish Cancer Society and The Velux Foundation (Veluxfonden).

The Danish Cancer Biobank (DCB) is acknowledged for biological material.

AHS

This work was supported by the Intramural Research Program of the NIH, National Cancer Institute, Division of Cancer Epidemiology and Genetics (Z01CP010119).

APCB

The Australian Prostate Cancer BioResource (APCB) was supported by The National Health and Medical Research Council, Enabling Grant [614296] and the Prostate Cancer Foundation of Australia.

The Australian Prostate Cancer BioResource (APCB) would like to acknowledge and sincerely thank the urologists, pathologists, coordinators, data managers, nurses and patient participants who have generously and altruistically supported the APCB.

W.D.T. was supported by The U.S. Department of Defense Prostate Cancer Research Program (Transformative Impact Award W81XWH-13-2-0093); The National Health and Medical Research Council (NHMRC) of Australia (1121057); the Prostate Cancer Foundation of Australia/Cancer Australia (1012337 and 1043482); Prostate Cancer Foundation of Australia/Movember Revolutionary Team Award; the Ray and Shirl Norman Cancer Research Trust.

ATBC

The ATBC Study is supported by the Intramural Research Program of the U.S. National Cancer Institute, National Institutes of Health, and by U.S. Public Health Service contract HHSN261201500005C from the National Cancer Institute, Department of Health and Human Services.

Canary PASS

PASS was supported by Canary Foundation and the National Cancer Institute's Early Detection Research Network) U01 CA086402)

CCI

This work was awarded by Prostate Cancer Canada and is proudly funded by the Movember Foundation - Grant # D2013-36.

The CCI group would like to thank David Murray, Razmik Mirzayans, and April Scott for their contribution to this work.

CHIPGECS
Orchid, Wuxi Second Hospital Research Funds, National Natural Science foundation of China for funding support to H Zhang (Grant No: 30671793 and 81072377), N Feng (Grant No: 81272831), SC Zhao (Grant No: 81072092 and 81328017)

This work was conducted on behalf of the CHIPGECS Consortia. We acknowledge the contribution of doctors, nurses and postgraduate research students at the CHIPGENCS sample collecting centers.

COH
SLN is partially supported by the Morris and Horowitz Families Endowed Professorship

COSM
The Swedish Research Council, the Swedish Cancer Foundation

CPCS1 & CPCS2
Department of Clinical Biochemistry, Herlev and Gentofte Hospital, Copenhagen University Hospital, Herlev Ringvej 75, DK-2730 Herlev, Denmark

We thank the participants and staff of the Copenhagen General Population Study for their important contributions.

CPS-II
The American Cancer Society funds the creation, maintenance, and updating of the Cancer Prevention Study II cohort.

We thank the CPS-II participants and Study Management Group for their invaluable contributions to this research. We would also like to acknowledge the contribution to this study from central cancer registries supported through the Centers for Disease Control and Prevention National Program of Cancer Registries, and cancer registries supported by the National Cancer Institute Surveillance Epidemiology and End Results program.

EPIC
The coordination of EPIC was financially supported by the European Commission (DG-SANCO) and the International Agency for Research on Cancer. The national cohorts (that recruited male participants) are supported by Danish Cancer Society (Denmark); German Cancer Aid, German Cancer Research Center (DKFZ), Federal Ministry of Education and Research (BMBF), Deutsche Krebshilfe, Deutsches Krebsforschungszentrum and Federal Ministry of Education and Research (Germany); the Hellenic Health Foundation (Greece); Associazione Italiana per la Ricerca sul Cancro-AIRC-Italy and National Research Council (Italy); Dutch Ministry of Public Health, Welfare and Sports (VWS), Netherlands Cancer Registry (NKR), LK Research Funds, Dutch Prevention Funds, Dutch ZON (Zorg Onderzoek Nederland), World Cancer Research Fund (WCRF), Statistics Netherlands (The Netherlands); Health Research Fund (FIS), PI13/00061 to Granada; , PI13/01162 to EPIC-Murcia), Regional Governments of Andalucía, Asturias, Basque Country, Murcia and Navarra, ISCIII RETIC (RD06/0020) (Spain); Swedish Cancer Society, Swedish Research Council and County Councils of Skåne and Västerbotten (Sweden); Cancer Research UK

(14136 to EPIC-Norfolk; C570/A16491 and C8221/A19170 to EPIC-Oxford), Medical Research Council (1000143 to EPIC-Norfolk, MR/M012190/1 to EPIC-Oxford) (United Kingdom). For information on how to submit an application for gaining access to EPIC data and/or biospecimens, please follow the instructions at http://epic.iarc.fr/access/index.php

EPICAP
The EPICAP study was supported by grants from Ligue Nationale Contre le Cancer, Ligue départementale du Val de Marne; Fondation de France; Agence Nationale de sécurité sanitaire de l'alimentation, de l'environnement et du travail (ANSES)

The EPICAP study group would like to thank all urologists, Antoinette Anger and Hasina Randrianasolo (study monitors), Anne-Laure Astolfi, Coline Bernard, Oriane Noyer, Marie-Hélène De Campo, Sandrine Margaroline, Louise N'Diaye, Sabine Perrier-Bonnet (Clinical Research nurses).

ERSPC
This study was supported by the DutchCancerSociety(KWF94-869,98-1657,2002-277,2006-3518, 2010-4800); The Netherlands Organisation for HealthResearch and Development (ZonMW-002822820,22000106,50-50110-98-311, 62300035), The Dutch Cancer Research Foundation(SWOP), and an unconditional grant from Beckman-Coulter-HybritechInc.

ESTHER
The ESTHER study was supported by a grant from the Baden Württemberg Ministry of Science, Research and Arts.

The ESTHER group would like to thank Hartwig Ziegler, Sonja Wolf, Volker Hermann, Heiko Müller, Karina Dieffenbach, Katja Butterbach for valuable contributions to the study.

FHCRC
The FHCRC studies were supported by grants R01-CA056678, R01-CA082664, and R01-CA092579 from the US National Cancer Institute, National Institutes of Health, with additional support from the Fred Hutchinson Cancer Research Center.

We thank all the men who participated in these studies.

Gene-PARE
The Gene-PARE study was supported by grants 1R01CA134444 from the U.S. National Institutes of Health, PC074201 and W81XWH-15-1-0680 from the Prostate Cancer Research Program of the Department of Defense and RSGT-05-200-01-CCE from the American Cancer Society. S.L.K. is supported by 1K07CA187546 from the U.S. National Cancer Institute.

HPFS
The Health Professionals Follow-up Study was supported by grants UM1CA167552, CA133891, CA141298, and P01CA055075.

We are grateful to the participants and staff of the Physicians’ Health Study and Health Professionals Follow-Up Study for their valuable contributions, as well as the following state cancer registries for their help: AL, AZ, AR, CA, CO, CT, DE, FL, GA, ID, IL, IN, IA, KY, LA, ME, MD, MA, MI, NE, NH, NJ, NY, NC, ND, OH, OK, OR, PA, RI, SC, TN, TX, VA, WA, WY.

IMPACT

The IMPACT study was funded by The Ronald and Rita McAulay Foundation, CR-UK Project grant (C5047/A1232), Cancer Australia, AICR Netherlands A10-0227, Cancer Australia and Cancer Council Tasmania, NIHR, EU Framework 6, Cancer Councils of Victorial and South Australia, Philanthropic donation to Northshore University Health System. We acknowledge support from the National Institute for Health Research (NIHR) to the Biomedical Research Centre at The Institute of Cancer Research and Royal Marsden Foundation NHS Trust.

We acknowledge the IMPACT study steering committee, collaborating centres and participants.

IPO-Porto

The IPO-Porto study was funded by Fundação para a Ciência e a Tecnologia (FCT; UID/DTP/00776/2013 and PTDC/DTP-PIC/1308/2014) and by IPO-Porto Research Center (CI-IPOP-16-2012 and CI-IPOP-24-2015). MC and MPS are research fellows from Liga Portuguesa Contra o Cancro, Núcleo Regional do Norte. SM is a research fellow from FCT (SFRH/BD/71397/2010).

We would like to express our gratitude to all patients and families who have participated in this study.

KULEUVEN

F.C. and S.J. are holders of grants from FWO Vlaanderen (G.0684.12N and G.0830.13N), the Belgian federal government (National Cancer Plan KPC_29_023), and a Concerted Research Action of the KU Leuven (GOA/15/017). TVDB is holder of a doctoral fellowship of the FWO.

Malaysia

The study was funded by the University Malaya High Impact Research Grant (HIR/MOHE/MED/35 to A.R.).

We thank all associates in the Urology Unit, University of Malaya, Cancer Research Malaysia (CRM) and the Malaysian Men's Health Initiative (MMHI).

MAYO

The Mayo group was supported by the US National Cancer Institute (R01CA72818)

MCCS

The Melbourne Collaborative Cohort Study (MCCS) cohort recruitment was funded by VicHealth and Cancer Council Victoria. The MCCS was further supported by Australian National Health and Medical Research Council grants 209057 and 396414 and by infrastructure provided by Cancer Council Victoria. Cases and their vital status were ascertained through the Victorian Cancer Registry and the Australian Institute of Health and Welfare, including the National Death Index and the Australian Cancer Database.

MCC-Spain

The study was partially funded by the "Accion Transversal del Cancer", approved on the Spanish Ministry Council on the 11th October 2007, by the Instituto de Salud Carlos III-FEDER (PI08/1770, PI09/00773-Cantabria, PI11/01889-FEDER, PI12/00265, PI12/01270, PI12/00715, PI15/00069), by the Fundación Marqués de Valdecilla (API 10/09), by the Spanish Association Against Cancer (AECC) Scientific Foundation and by the Catalan Government DURSI grant 2009SGR1489. Samples: Biological samples were stored at the Parc de Salut MAR Biobank (MARBiobanc; Barcelona) which is supported by Instituto de Salud Carlos III FEDER (RD09/0076/00036). Also sample collection was supported by the Xarxa de Bancs de Tumors de Catalunya sponsored by Pla Director d'Oncologia de Catalunya (XBTC). ISGlobal is a member of the CERCA Programme, Generalitat de Catalunya.

We acknowledge the contribution from Esther Gracia-Lavedan in preparing the data. We thank all the subjects who participated in the study and all MCC-Spain collaborators.

MEC

The MEC was supported by NIH grants CA63464, CA54281, CA098758, and CA164973.

MOFFITT

The Moffitt group was supported by the US National Cancer Institute (R01CA128813, PI: J.Y. Park).

Oslo

CONOR was supported by grants from the Nordic Cancer Union, the Swedish Cancer Society (2012/823) and the Swedish Research Council (2014/2269).

The authors wish to acknowledge the services of CONOR, the contributing research centres delivering data to CONOR, and all the study participants.

PCMUS

The PCMUS study was supported by the Bulgarian National Science Fund, Ministry of Education and Science (contract DOO-119/2009; DUNK01/2-2009; DFNI-B01/28/2012) with additional support from the Science Fund of Medical University - Sofia (contract 51/2009; 8I/2009; 28/2010;).

PHS

The Physicians' Health Study was supported by grants CA34944, CA40360, CA097193, HL26490 and HL34595.

We are grateful to the participants and staff of the Physicians' Health Study and Health Professionals Follow-Up Study for their valuable contributions, as well as the following state cancer registries for their help: AL, AZ, AR, CA, CO, CT, DE, FL, GA, ID, IL, IN, IA, KY, LA, ME, MD, MA, MI, NE, NH, NJ, NY, NC, ND, OH, OK, OR, PA, RI, SC, TN, TX, VA, WA, WY.

PLCO

This PLCO study was supported by the Intramural Research Program of the Division of Cancer Epidemiology and Genetics, National Cancer Institute, NIH

The authors thank Drs. Christine Berg and Philip Prorok, Division of Cancer Prevention at the National Cancer Institute, the screening center investigators and staff of the PLCO Cancer Screening Trial for their contributions to the PLCO Cancer Screening Trial. We thank Mr. Thomas Riley, Mr. Craig Williams, Mr. Matthew Moore, and Ms. Shannon Merkle at Information Management Services, Inc., for their management of the data and Ms. Barbara O'Brien and staff at Westat, Inc. for their contributions to the PLCO Cancer Screening Trial. We also thank the PLCO study participants for their contributions to making this study possible.

PRAGGA

PRAGGA was supported by Programa Grupos Emergentes, Cancer Genetics Unit, CHUVI Vigo Hospital, Instituto de Salud Carlos III, Spain.

PRAGGA wishes to thank Victor Muñoz Garzón, Manuel Enguix Castelo, Sara Miranda Ponte, Carmen M Redondo, Manuel Calaza, Francisco Gude Sampedro, Joaquín González-Carreró and the staff of the Department of Pathology and Biobank of University Hospital Complex of Vigo, Instituto de Investigación Sanitaria Galicia Sur (IISGS), SERGAS, Vigo, Spain; Máximo Fraga, José Antúnez and the Biobank of University Hospital Complex of Santiago, Santiago de Compostela, Spain; and Maria Torres, Angel Carracedo and the Galician Foundation of Genomic Medicine.

PROCAP

PROCAP was supported by the Swedish Cancer Foundation (08-708, 09-0677).

We thank and acknowledge all of the participants in the PROCAP study. We thank Carin Cavalli-Björkman and Ami Rönnberg Karlsson for their dedicated work in the collection of data. Michael Broms is acknowledged for his skilful work with the databases. KI Biobank is acknowledged for handling the samples and for DNA extraction. We acknowledge The NPCR steering group: Pär Stattin (chair), Anders Widmark, Stefan Karlsson, Magnus Törnblom, Jan Adolfsson, Anna Bill-Axelson, Ove Andrén, David Robinson, Bill Pettersson, Jonas Hugosson, Jan-Erik Damber, Ola Bratt, Göran Ahlgren, Lars Egevad, and Roy Ehrnström.

PROFILE

We would like to acknowledge the support of the Ronald and Rita McAulay Foundation and Cancer Research UK. We also acknowledge support from the National Institute for Health Research (NIHR) to the Biomedical Research Centre at The Institute of Cancer Research and Royal Marsden Foundation

NHS Trust

We acknowledge the Profile study steering committee and participants.

PROGReSS

The PROGReSS study is funded by grants from the Spanish Ministry of Health (INT15/00070; INT16/00154; FIS PI10/00164, FIS PI13/02030; FIS PI16/00046); the Spanish Ministry of Economy and Competitiveness (PTA2014-10228-I) and Fondo Europeo de Desarrollo Regional (FEDER 2007-2013). LF was funded by European Union Marie Sklodowska-Curie Individual Fellowship (MSCA-IF-2014-EF-656144)

ProMPT & ProtecT

ProtecT would like to acknowledge the support of The University of Cambridge, Cancer Research UK. Cancer Research UK grants [C8197/A10123] and [C8197/A10865] supported the genotyping team. We would also like to acknowledge the support of the National Institute for Health Research which funds the Cambridge Bio-medical Research Centre, Cambridge, UK. We would also like to acknowledge the support of the National Cancer Research Prostate Cancer: Mechanisms of Progression and Treatment (PROMPT) collaborative (grant code G0500966/75466) which has funded tissue and urine collections in Cambridge. We are grateful to staff at the Welcome Trust Clinical Research Facility, Addenbrooke's Clinical Research Centre, Cambridge, UK for their help in conducting the ProtecT study. We also acknowledge the support of the NIHR Cambridge Biomedical Research Centre, the DOH HTA (ProtecT grant) and the NCRI / MRC (ProMPT grant) for help with the bio-repository. The UK Department of Health funded the ProtecT study through the NIHR Health Technology Assessment Programme (projects 96/20/06, 96/20/99). The ProtecT trial and its linked ProMPT and CAP (Comparison Arm for ProtecT) studies are supported by Department of Health, England; Cancer Research UK grant number C522/A8649, Medical Research Council of England grant number G0500966, ID 75466 and The NCRI, UK. The epidemiological data for ProtecT were generated though funding from the Southwest National Health Service Research and Development. DNA extraction in ProtecT was supported by USA Dept of Defense award W81XWH-04-1-0280, Yorkshire Cancer Research and Cancer Research UK. The authors would like to acknowledge the contribution of all members of the ProtecT study research group. Richard Martin was supported by a Cancer Research UK Programme Grant (C18281/A19169) and the National Institute for Health Research Bristol Nutrition Biomedical Research Centre based at University Hospitals Bristol NHS Foundation Trust and the University of Bristol. The views and opinions expressed therein are those of the authors and do not necessarily reflect those of the Department of Health of England. The bio-repository from ProtecT is supported by the NCRI (ProMPT) Prostate Cancer Collaborative and the Cambridge BMRC grant from NIHR. We thank the National Institute for Health Research, Hutchison Whampoa Limited, the Human Research Tissue Bank (Addenbrooke's Hospital), and Cancer Research UK. The authors would like to thank those men with prostate cancer and the subjects who have donated their time and their samples to the Cambridge Biorepository, which were used in this research. We also would like to acknowledge to support of the research staff in S4 who so carefully curated the samples and the follow-up data (Jo Burge, Marie Corcoran, Anne George, and Sara Stearn).

QLD

The QLD research is supported by The National Health and Medical Research Council (NHMRC) Australia Project Grants [390130, 1009458] and NHMRC Career Development Fellowship and Cancer Australia PdCCRS funding to J Batra.

The QLD team would like to acknowledge and sincerely thank the urologists, pathologists, data managers and patient participants who have generously and altruistically supported the QLD cohort.

RAPPER

RAPPER is funded by Cancer Research UK [C1094/A11728; C1094/A18504] and Experimental Cancer Medicine Centre funding [C1467/A7286]

The RAPPER group thank Rebecca Elliott for project management.

SEARCH

SEARCH is funded by a programme grant from Cancer Research UK [C490/A10124] and supported by the UK National Institute for Health Research Biomedical Research Centre at the University of Cambridge. The University of Cambridge has received salary support in respect of PP from the NHS in the East of England through the Clinical Academic Reserve.

SFPCS

SFPCS was funded by the California Cancer Research Fund grant 99-00527V-10182.

SNP_Prostate_Ghent

The study was supported by the National Cancer Plan, financed by the Federal Office of Health and Social Affairs, Belgium.

SPAG

Wessex Medical Research

Hope for Guernsey, MUG, HSSD, MSG, Roger Allsopp

STHM2

STHM2 was supported by grants from The Strategic Research Programme on Cancer (StratCan), Karolinska Institutet; the Linné Centre for Breast and Prostate Cancer (CRISP, number 70867901), Karolinska Institutet; The Swedish Research Council (number K2010-70X-20430-04-3) and The Swedish Cancer Society (numbers 11-0287 and 11-0624); Stiftelsen Johanna Hagstrand och Sigfrid Linnérs minne; Swedish Council for Working Life and Social Research (FAS), number 2012-0073.

The authors acknowledge the Karolinska University Laboratory, Aleris Medilab, Unilabs and the Regional Prostate Cancer Registry for performing analyses and help to retrieve data. Carin Cavalli–Björkman and Britt-Marie Hune for their enthusiastic work as research nurses. Astrid Björklund for skilful data management. We wish to thank the BBMRI.se biobank facility at Karolinska Institutet for biobank services.

SWOG-PCPT

PCPT is funded by Public Health Service grants U10CA37429 and 5UM1CA182883 from the National Cancer Institute.

The authors thank the site investigators and staff and, most importantly, the participants from PCPT who donated their time to this trial.

SWOG-SELECT

SELECT is funded by Public Health Service grants U10CA37429 and 5UM1CA182883 from the National Cancer Institute.

The authors thank the site investigators and staff and, most importantly, the participants from SELECT who donated their time to this trial.

TAMPERE

The Tampere (Finland) study was supported by the Academy of Finland (251074), The Finnish Cancer Organisations, Sigrid Juselius Foundation, and the Competitive Research Funding of the Tampere University Hospital (X51003). The PSA screening samples were collected by the Finnish part of ERSPC (European Study of Screening for Prostate Cancer).

TAMPERE would like to thank Riina Liikanen, Liisa Maeaettaenen and Kirsi Talala for their work on samples and databases.

Toronto

Prostate Cancer Canada Movember Discovery Grant (D2013-17) to RJH; Canadian Cancer Society Research Institute Career Development Award in Cancer Prevention (2013-702108) to RJH

UKGPCS

UKGPCS would also like to thank the following for funding support: The Institute of Cancer Research and The Everyman Campaign, The Prostate Cancer Research Foundation, Prostate Research Campaign UK (now Prostate Action), The Orchid Cancer Appeal, The National Cancer Research Network UK, The National Cancer Research Institute (NCRI) UK. We are grateful for support of NIHR funding to the NIHR Biomedical Research Centre at The Institute of Cancer Research and The Royal Marsden NHS Foundation Trust. UKGPCS should also like to acknowledge the NCRN nurses, data managers and Consultants for their work in the UKGPCS study.

UKGPCS would like to thank all urologists and other persons involved in the planning, coordination, and data collection of the study.

ULM

The Ulm group received funds from the German Cancer Aid (Deutsche Krebshilfe).

UTAH

The Keith and Susan Warshaw Fund, C. S. Watkins Urologic Cancer Fund and The Tennity Family Fund supported the Utah study. The project was supported by Award Number P30CA042014 from the National Cancer Institute

WUGS/WUPCS

WUGS would like to thank the following for funding support: The Anthony DeNovi Fund, the Donald C. McGraw Foundation, and the St. Louis Men’s Group Against Cancer.
