# Supplementary material for: Germline variation at 8q24 and prostate cancer risk in men of European ancestry
Source: Nat Commun. 2018 Nov 5;9:4616. doi: 10.1038/s41467-018-06863-1 (PMC6218483; doi:10.1038/s41467-018-06863-1)
Supplement: Supplementary file 7 — Description of Additional Supplementary Files [file 41467_2018_6863_MOESM7_ESM.pdf]

Description of Additional Supplementary Files

File Name: Supplementary Data 1
Description: Pairwise LD between genetic variants independently associated with PCa risk in the current study and previously published PCa risk variants at 8q24.

File Name: Supplementary Data 2
Description: Variants in the 95% credible set and correlated variants.

File Name: Supplementary Data 3
Description: Functional annotation for variants in the 95% credible set and correlated variants.

File Name: Supplementary Data 4
Description: Proportion of familial relative risk (FRR) explained by the 175 established PCa susceptibility loci including the 12 risk variants at 8q24.
